# Colonization history of snow algae on Hawai‘i island

**DOI:** 10.1093/ismejo/wraf197

**Published:** 2025-09-05

**Authors:** Takahiro Segawa, Nozomu Takeuchi, Ryo Matsuzaki, Takahiro Yonezawa, Kenji Yoshikawa

**Affiliations:** Center for Life Science Research, University of Yamanashi, 1110, Shimokato, Chuo, Yamanashi, 409-3898, Japan; Department of Earth Sciences, Graduate School of Science, Chiba University, 1-33 Yayoi-cho, Inage-ku, Chiba, 263-8522, Japan; Department of General Education, Faculty of Engineering, Osaka Institute of Technology, 5-16-1 Omiya, Asahi-ku, Osaka, 535-8585, Japan; Graduate School of Integrated Sciences for Life, Hiroshima University, 1-4-4 Kagamiyama, Higashi-Hiroshima City, Hiroshima, 739-8528, Japan; Water and Environmental Research Center, Institute of Northern Engineering, University of Alaska Fairbanks, Fairbanks, AK 99775, United States

**Keywords:** snow algae, red snow, biogeography, evolution, cryosphere, Hawai‘i Island

## Abstract

Red-pigmented snow algae are cold-adapted (including cryophilic) photosynthetic microbes commonly found in polar and alpine snowpacks worldwide, but their dispersal across isolated cryospheres remains poorly understood. We report the occurrence of snow algae on Maunakea, Hawai‘i, the most isolated cryosphere in the world, during an unusually prolonged summer snow retention event in 2023 associated with La Niña conditions. Red-pigmented algal cells were observed in snow samples collected during this event. ITS2 amplicon sequencing identified two major Chlorophyta groups: the cosmopolitan *Sanguina* group and the endemic *Chloromonadinia* snow group. The cosmopolitan *Sanguina* group disperses into Hawai‘i from other cryospheres under present climate conditions, whereas the endemic *Chloromonadinia* assemblage shows multiple arrivals, with the largest Hawaiian clade indicating colonization between ~253 and 130 ka, overlapping the Pohakuloa glaciation (MIS 6) when Maunakea’s summit was ice-capped. This study shows how specific climate conditions, such as glaciation, provided long-term habitats that enabled the establishment of distinct snow algae lineages, highlighting the timing, and processes of their dispersal as shaped by glaciation and climate change.

## Introduction

Red snow caused by microbial activity has long intrigued scientists [1, 2]. This phenomenon, observed across various cold regions worldwide, continues to be of scientific interest due to its ecological and evolutionary significance, as well as its impact on albedo and climate feedback [3–7]. Snow algae are cold-adapted photosynthetic microbes that produce red, green, or orange pigments, and are one of the primary causes of colored snow in polar and alpine snowpacks [8, 9]. They play a crucial role in snow and ice ecosystems by influencing the albedo of snow, potentially accelerating snowmelt [10, 11]. Despite the discontinuous distribution of the terrestrial cryosphere regions, the broad distribution across polar and mid-latitude regions of snow algae, along with their functional roles in primary production and albedo reduction, underscores their ecological significance [12, 13].

In polar regions, snow algae comprise a limited number of cosmopolitan phylotypes and a highly diverse range of endemic phylotypes (unique ITS2 genotypes) [6]. A recent study of the genus *Raphidonema* revealed that endemic phylotypes originated from ancestral cosmopolitan phylotypes, suggesting that regional diversity in this group could arise through microevolution driven by geographic isolation and local adaptation [14]. This raises important questions about the global dispersal of snow algae and whether cosmopolitan and endemic phylotypes follow distinct dispersal processes, particularly influenced by glacial–interglacial climate cycles. Investigating these processes in connection with past climate fluctuations is crucial for understanding the biogeographical patterns that govern their distribution across the global cryosphere.

The Hawaiian Islands provide a unique opportunity to address these questions. Located in the heart of the central Pacific Ocean, the islands are ~3900 km from the west coast of North America, the nearest continent. This geographical isolation has led to a highly unique environment and ecosystem, with ~77%–90% of terrestrial species being endemic to the islands [15–17]. Therefore, studying snow algae in these islands may offer valuable insights into their global dispersal and evolutionary mechanisms in isolated cryospheres.

On Hawai‘i Island, Maunakea and Maunaloa both exceed 4000 m a.s.l. and provide potential habitats for snow algae. Rare snowfalls also occur on Haleakalā (3055 m) on Maui and Hualalai (2521 m) on Hawai‘i Island, but snow cover is more consistent on Maunakea and Maunaloa. Despite the occurrence of such snow cover, the presence of snow algae in Hawai‘i has not previously been reported.

In the present climate, snow conditions on Maunakea are closely tied to El Niño–Southern Oscillation (ENSO) events [18]. In El Niño years, there is little snowfall and the snowpack melts within a month. In neutral or La Niña years, snowfall is more abundant, with winter storms occasionally depositing snow that persists into spring and early summer, particularly during La Niña years when cooler air temperatures and increased precipitation allow the snowpack to remain until March or April [19]. As solar radiation intensifies toward the summer solstice, the remaining snow melts rapidly [20]. In such conditions, the extended presence of snow creates an environment conducive to snow-algae proliferation on the snow surface, providing sufficient time for their growth [21].

**Figure 1:**
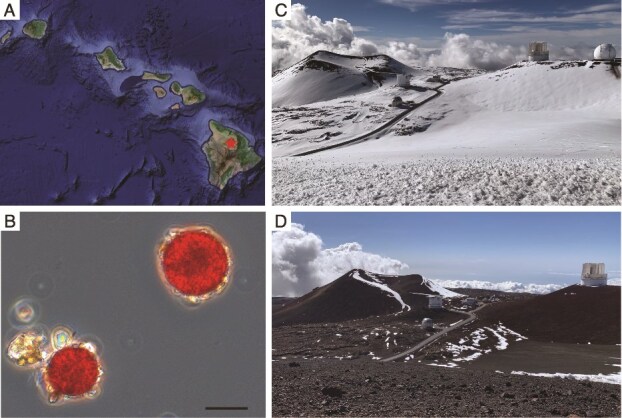
Location of sampling site and the presence of red snow-causing algae. (A) Location of Maunakea (indicated by a star) on the island of Hawai‘i. (B) Representative microscopic image of dominant red-pigmented snow algae in snow samples from 23 July 2023. Scale bar, 20 μm. (C,D) Representative photographs of red snow on Maunakea on January 21, 2021 (C) and March 26, 2021 (D).

Here, we report the occurrence of snow algae that produce red pigments and cause visible red snow (hereafter, red snow algae) on Hawai‘i Island (Fig. 1). In 2023, the snowpack on Maunakea persisted until the end of July due to strong La Niña conditions, likely creating a favorable environment for algae growth. Using phylogenetic analysis of sequences of the internal transcribed spacer 2 (ITS2) region of nuclear ribosomal DNA, we investigated the presence, distribution, and evolutionary history of red snow algae on Hawai‘i Island and compared them with populations in polar and mid-latitude regions. This study aimed to determine whether these algae are recent atmospheric immigrants or long-isolated lineages that arrived during earlier climatic events and have since undergone phylogeographic differentiation within the Islands.

## Materials and methods

### Snow samples

Surface snow samples were collected from Maunakea (19.82°N, 155.47°W) on Hawai‘i Island on 26 March and 17 April 2021, and on 5 February, 17 April, 30 June, 18 July, and 20 July 2023. This allowed for comparison of snow cover observations between the two years, with particular emphasis on the unusual snow retention observed in 2023. Fresh snow was also collected on 5 February 2023. Specifically, samples were collected on the north face of Mt. Pu’u Poliahu. For each sampling event, 1–2 cm of surface snow was collected from three randomly selected points, and ~0.5–6 l of snow were collected per sampling event. Samples were collected into sterile plastic bags (Whirl-Pak, Nasco, United States) using a sterilized stainless-steel scoop, with sterile gloves and masks worn during the collection process. Samples were kept frozen during transport to the University of Yamanashi, Japan, and then stored at −80°C until use.

### Determination of algae concentration and cell size

Samples were stored at −80°C to preserve cell morphology and thawed immediately prior to use. From the melted snow samples, 100–1000 μl of water were filtered through a PTFE membrane filter (pore size 0.22 μm, FHLC01300; Millipore), and the number of algae on the filter was counted using a fluorescence microscope (IX81; Olympus, Tokyo, Japan). Counting was conducted five times per sample. Mean results were used to obtain the cell concentration (cells/ml) of each sample. Cell size measurements were conducted using samples collected on 18 July 2023. Although red-pigmented algal cells were consistently observed under the microscope, the snow surface did not display visible red coloration. The pigmentation was only faintly discernible in close-up photographs of the snow surface (Supplementary Fig. S1), and not apparent to the naked eye in the field.

### High-performance liquid chromatography

The meltwater of the samples (35 ml) was filtered through a glass fiber filter (Whatman Glass Microfiber Filters, GF/F, 25 mm). The filter was then placed in 1 ml of N,N-dimethylformamide (DMF, 99.5%) and left in the refrigerator for at least 24 h. The extracted pigments were stored at −20°C before analysis. A Prominence Series instrument (Shimadzu, Japan) was used, including a liquid chromatograph (LC-20 AD, Shimadzu), fluorescence detector (RF-20AXS, Shimadzu), diode array detector (SPD-M30A, spectra from 190 to 700 nm, Shimadzu), solvent degasser, autosampler unit set at 4°C, column thermostat set at 60°C, and a reversed-phase C8 column (Phenomenex LUNA-C8 [2], 3 μm, 150 × 4.6 mm). Solvent A was a 3:7 (v/v) solution of 28 mM tetrabutylammonium acetate (1.0 M in water, Sigma-Aldrich) and methanol (HPLC grade, Fujifilm-Wako); solvent B was methanol alone. A 2:1 (v/v) mixed solution of solvent A and filtered sample (total 400 μL) was injected and analyzed at a flow rate of 1 ml /min. Fluorescence at 735 nm (excitation 450 nm) was used to quantify chlorophylls *a* and *b*, and absorption at 450/510 nm was used for other carotenoid pigments. Each pigment is shown as the percentage of the total mass of major chlorophyll and carotenoid pigments quantified in this analysis.

### DNA extraction

All work prior to genome library amplification was conducted in a clean room at the University of Yamanashi, Japan. For each sampling date, three stored snow samples collected from different points on the same sampling date were combined into a single pooled sample to reduce sampling bias and obtain a representative profile of snow algal community. Water samples (200–4000 ml from February to June, 2–10 ml from July) were filtered through a sterile 0.22 μm membrane filter (Nalgene Analytical Filter Unit, Thermo Scientific, United States) to collect microorganisms. Membrane filters were transferred to 2- ml Matrix-E tubes (Qbiogene, United States) with 600 μl extraction buffer as described [22]. Two sequential homogenizations were carried out with a multibead shocker (Yasui Kikai, Japan) at 1000 rpm for 30 s to remove microorganisms and then at 2500 rpm for 30 s to disrupt them. The homogenized samples were incubated with proteinase K (Roche) for at least 4 h at 55°C with agitation. Samples were extracted with 1.15 M NaCl and ½ vol. of chloroform/octanol (24:1) for 15 min at room temperature with gentle agitation. The mixtures were centrifuged at 12 000 × *g* for 5 min, and the aqueous phase was collected and transferred to separate microtubes. Further purification steps followed a previously described protocol [23]. The supernatants were combined with 10 ml Binding Buffer B and processed using silica spin column pre-assembled with a volume extender (High Pure Viral Nucleic Acid Large Volume kit, Roche). All DNA extractions were conducted on a class 100 clean bench (Nippon Ikakikai Seisakusyo, Japan), and subsequent procedures were carried out on another class 100 clean bench to minimize contamination.

### ITS2 region amplicon sequencing

The ITS2 region was amplified by PCR using primers c and b (5′-GCATCGATGAAGAACGCAGC-3′ and 5′-GGGATCCATATGCTTAAGTTCAGCGGGT-3′, respectively [24]), with Illumina overhang adapter sequences at the 5′ ends. Each PCR mixture (25 μl) contained 1× KAPA HiFi HS ReadyMix (Kapa Biosystems, USA), 0.2 μm of each primer, and 2–4 μl template DNA. The PCR cycling conditions were as follows: initial denaturation at 95°C for 3 min; 25–35 cycles at 95°C for 30 s, 50°C for 30 s, and 72°C for 60 s; and final extension at 72°C for 5 min. Amplicons with overhang adapter sequences were generated in triplicate, and each amplicon was pooled before index PCR. PCR products were labeled with two sample-specific indices each containing a sample-unique index [25] and adapter sequences at their 5′ ends (Nextera XT Index Kit v2, Illumina). The PCR mixture (10 μl contained 1× KAPA HiFi HS ReadyMix, 2 μl each of forward and reverse primers, and 1 μl of recovered PCR products. PCR was performed under the following cycling conditions: 95°C for 3 min; eight cycles of 95°C for 30 s, 55°C for 30 s, and 72°C for 60 s; and final extension at 72°C for 5 min. After agarose gel electrophoresis, PCR products were excised and purified using the NucleoSpin Gel and PCR Clean-up kit (Macherey-Nagel, Germany). Tagged amplicons were mixed with PhiX control DNA at a ratio of 80:20 and sequenced (2 × 300 bp, paired-end) on a MiSeq System (Illumina) using the MiSeq Reagent Kit v3.

### Quantitative PCR analysis

Quantitative PCR (qPCR) assays were developed to specifically detect and quantify ASV1 (*Chloromonadinia* snow group) and ASV2 (*Sanguina* group 2). Primer and TaqMan probe sets were designed using PrimerExpress software v3.0 (Thermo Fisher Scientific). The primer and probe sequences were as follows: ASV1: forward primer, TGGTGAGGTCTGCCAAAGTG; reverse primer, CAGTCATTGCTGACCGAAGKA; probe, 6-FAM- AAGTAGCGC/ZEN/TCCGAGCATCGCCT—IABkFQ.

ASV2: forward primer, TTCGGCCAACAGCATGTCT; reverse primer, ATCCGCCCAGATCATCAACA; probe, 6-FAM- CCTCAGCGT/ZEN/CGGGTTAATTCTCGCC—IABkFQ. Synthetic gBlocks Gene Fragments (Integrated DNA Technologies), each containing the target sequence of ASV1 or ASV2, were synthesized separately and used as positive controls to construct standard curves (10^0^ to 10^6^ copies μl^−1^) for each assay. All qPCR assays were performed in triplicate using TaqMan Fast Advanced Master Mix (Thermo Fisher Scientific) on a QuantStudio 1 Real-Time PCR System (Applied Biosystems). The thermal cycling protocol consisted of an initial denaturation at 95°C for 20 s, followed by 40 cycles of 95°C for 1 s, 55°C for 20 s, and 60°C for 20 s. Quantification was based on standard curves derived from serial dilutions of the gBlocks.

### Bioinformatics

Sequence data were analyzed using DADA2 v. 1.22 [26]. The generated ITS2 sequence reads were denoised and clustered into ASVs, which represent distinct sequences rather than clusters based on sequence similarity. Taxonomic assignments of unique ITS2 sequences were conducted by a BLASTn search [27] with a top-hit E-value of <1e^−8^, identity of >90%, and alignment length of >200 bp against (i) the UNITE fungal ITS2 sequence database v9.0 [28], (ii) Viridiplantae ITS2 sequences obtained from NCBI, and (iii) unique ITS2 sequences of snow algae previously detected in red snow [14]. Sequences below the 90% identity threshold were removed to exclude potential nontarget sequences. The retained ITS2 sequences were then clustered at 98% nucleotide identity using the furthest neighbor algorithm in Mothur 1.47.1 [29] to reduce redundancy and facilitate phylotype-based diversity analysis. Using the unique sequences of the ITS2 region detected from each geographical region, we divided the distribution into two types: widespread (including cosmopolitans) and endemics, based on their observed geographic distribution. In this context, phylotypes are defined as unique ITS2 genotypes represented by DADA2-derived ASVs or unique ITS2 sequences from our previous study. Cosmopolitan types are distributed at both poles and the middle latitudes (Hawai‘i, Antarctica, Svalbard, Greenland, Alaska, and mid-latitude regions).

### Groups based on the secondary structure of the ITS2 region

To estimate the diversity of snow algae within snow samples, ITS2 region sequences were classified at the species level according to the genetic species concept based on structural differences in the ITS2 region [30]. Hereafter, these unique sequences are defined as “phylotypes” and ITS2 sequences with ≥98% nucleotide sequence identity as OTUs. The 5.8S–28S rRNA interaction region in each OTU sequence was annotated using the web interface for hidden Markov model annotations [31] with the ITS2 database [32]. Secondary structures for ITS2 were predicted using Centroidfold [33] and RNAfold WebServer [34] and were refined manually. We confirmed that the ITS2 secondary structures of the OTUs examined in this study contained common structural hallmarks of eukaryotic ITS2 sequences: four helices with a U–U mismatch in helix II and a YGGY motif on the 5′ side of helix III near the apex [35, 36]. The ITS2 sequences of the OTUs were then compared with published sequences using BLASTn in the NCBI nonredundant nucleotide (nt) database. Based on the BLASTn results, the OTUs were classified into two chlorophycean and nine trebouxiophycean groups (Supplementary Tables S1 and S2). Within each group, species boundaries among OTUs were estimated based on the compensatory base change near the apex of helix III encompassing the YGGY motif (the most conserved region of the ITS2 secondary structure in eukaryotes) [37]. Although not universally definitive [38], the presence of a compensatory base change in the region has been shown to correlate with biological species separation in many eukaryotic lineages, especially within the green algal order Chlamydomonadales [30] which includes numerous snow algae (e.g. *Sanguina, Rosetta,* and *Chloromonadinia* snow group).

Chimeric clusters were primarily removed by BLASTn to the NCBI nt database. After grouping the sequences based on the secondary structures, possible chimeric sequences were checked manually and deleted. The unique sequences and OTUs of the algal ITS2 sequence clusters were identified from taxonomic assignment results, and the remaining unique sequences and OTUs that were not assigned to algae were discarded.

### Phylogenetic analysis

The unique ITS2 sequences of the C*hloromonadinia* snow group and *Sanguina* group from this study, together with the ITS2 sequences of snow algae in Antarctica, the Arctic, and mid-latitude regions reported by previous studies [6, 14] and those from the NCBI nt database (BLASTn searches were performed to identify the closest sequences in the NCBI nt database, i.e. those with >98.0% similarity), were aligned using MAFFT v. 7.518 [39]. A maximum likelihood tree was inferred using IQ-TREE v. 1.6.12 [40] with the best-fit model (GTR + F + I + G4 model). Standard bootstrap analysis was carried out with 1000 replications to evaluate the confidence of the tree topology.

### Estimating coalescence times

Coalescence times of snow algae were estimated by the Bayesian hierarchical model using BEAST v. 1.10.4 [41]. The alignment used for the maximum likelihood tree inference was also used for this analysis. Because it is very difficult to provide the time constraint for the internal nodes within the snow algal phylogeny based on geological or paleomicrobiological evidence, ^14^C dating and pollen dating of ice core samples converted to calendar ages were used for tip-dating analysis (37–8127 years BP) [14]. These ancient ITS2 sequences were not assumed to represent direct ancestors of the Hawai‘i lineages, but were incorporated as dated tips to inform the overall temporal scaling of the tree. Phylogenetic analysis of the *Chloromonadinia* snow group was conducted using 182 ASVs from ice cores as well as 104 ASVs from modern snow samples including those from Hawai‘i. Phylogenetic analysis of the *Sanguina* group was conducted using 66 ASVs from ice cores as well as 125 ASVs from modern snow samples including those from Hawai‘i. Coalescence times of the *Chloromonadinia* snow group and *Sanguina* group were estimated independently. The uncorrelated log-normal relaxed clock model was applied and coalescent processes with constant population size were assumed for the tree shape prior model. The HKY + I + Γ model was used for the nucleotide substitution model. A Markov chain Monte Carlo (MCMC) simulation was conducted using a length of 1 × 10^9^ generations, and trees were sampled every 1 × 10^5^ generations. The convergence of each parameter was confirmed by checking that every effective sample size was >200 using TRACER v. 1.7 (http://tree.bio.ed.ac.uk/software/tracer/).

### Demographic analysis

We estimated the demographic histories of cosmopolitans and endemics for each of Hawai‘i clades 1–3 based on the Bayesian skyline plot [42] using BEAST v. 1.10.4 [43]. To account for different evolutionary modes of nucleotide substitution rates among clades, the strict molecular clock model was evaluated for each clade using the likelihood ratio test (LRT) with PAML v. 4.9. Coalescence times for Hawai‘i clades 2 and 3 were estimated using the strict clock model, as it was not rejected by the likelihood ratio test (LRT; *P* > .05) in either clade. The uncorrelated log-normal relaxed molecular clock model was used for Hawai‘i clade 1, in which the strict molecular clock model was rejected. Because these data did not include ice core samples, the tip-dating method could not be used. Therefore, the time to most recent common ancestor of each clade estimated for the entire *Chloromonadinia* snow group or *Sanguina* group was applied as a prior probability distribution of Tree Root Height. The HKY + I + Γ model was used for the nucleotide substitution model. A Markov chain Monte Carlo was conducted using 1 × 10^9^ generations, and trees were sampled every 1 × 10^5^ generations.

## Results

### Maunakea snowpack and red snow algae (2021 and 2023)

Snow cover duration and algal presence were compared between 2021 and 2023, based on samples collected in both years. In 2023, the snowpack on Maunakea persisted into late July, an unusually long-lasting event in recent decades. According to historical records, residual snow lasting into July has only been observed four times over the past 50 years (1975, 1989, 1990, and 2023). For the first time in at least 33 years, snow remained on Maunakea through mid-summer, with the snow patch reaching a size of ~350 m^2^ in July. Typically, snow on Maunakea disappears by early May. However, in 2021 it remained until late April following a major snowfall event in late January.

Red-pigmented snow algae were detected in samples collected in April 2021 and in June and July 2023, but not in samples from March or April 2023 (Supplementary Fig. S2B). This observation indicates that algal presence was associated with snow cover duration, with detectable blooms occurring only in the later stages of snow persistence.

### Snow algae and their phylotypes in the Maunakea snowpack

Red-pigmented spherical cells were observed by microscopy in snow samples collected in April 2021 and June–July 2023 (Fig. 1). The cell diameter ranged from 12 to 74 μm with multiple peaks, suggesting the presence of various species. One of the peaks corresponded to the size of *Sanguina* species (8–39 μm), which are the typical red-pigmented snow algae observed in other regions [44, 45] (Supplementary Fig. S2A). Cell concentrations ranged from 87 to 12 300 cells/ ml in April 2021 and June–July 2023 (Supplementary Fig. S2B).

The samples taken in June and July 2023 underwent algal pigment analysis using high-performance liquid chromatography (HPLC). This analysis revealed that astaxanthin was the primary pigment responsible for the red color. Seasonal variation in pigment composition from June to July showed that the abundance of astaxanthin diesters, which have been reported to be abundant in the *Chloromonadinia* snow group [13], decreased while astaxanthin monoesters increased, indicating a shift in the algal community to dominance of the *Sanguina* group as the summer progressed (Supplementary Fig. S3). These seasonal pigment changes highlight a dynamic ecosystem, with different algal groups dominating at different stages of snow persistence.

**Figure 2:**
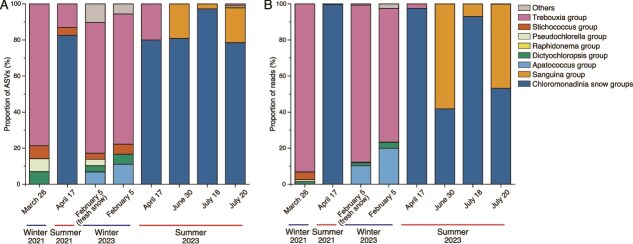
Algal taxonomic composition in Maunakea snow samples based on ITS2 sequences. The bar chart presents the average community composition for each sampling date, categorized into the eight major ITS2 groups indicated plus low-abundance groups (grouped as “others”). Proportions (%) of ASV sequences (A) and sequencing reads (B) are shown.

To assess the diversity of snow algae, we performed amplicon analysis of the ITS2 region, which revealed 328 amplicon sequence variants (ASVs) of Chlorophyta. Phylogenetic analysis showed a broad diversity of lineages in the Hawai‘i snow patch (Fig. 2, Supplementary Figs. S4–S8, Supplementary Tables S1 and S2). One group was *Trebouxia*, a genus typically associated with lichens rather than snow algae [7]; it was observed only in newly fallen snow or snow that had been on the ground for > 2 months. In contrast, the *Chloromonadinia* snow group (79% of total ASVs, 66% of total reads) and the *Sanguina* group (7% of total ASVs, 26% of total reads) dominated in snow samples that had persisted for more than two months during summer. The *Raphidonema* group was also detected, although it represented a minor fraction of the community (0.3% of total ASVs, 0.01% of total reads).

In June and July 2023, *Chloromonadinia* snow groups 28 and 32 and *Sanguina* group 2 accounted for 86% of total reads, indicating their dominance within the snow algal community during the summer snowmelt period. Seasonal change in the algal community was apparent, as the *Chloromonadinia* snow groups were detected in April 2021 and from April to July 2023, whereas the *Sanguina* group 2 appeared only in snow that had persisted into June and July 2023, when prolonged melt conditions likely increased water availability. It is also possible that *Sanguina* group 2 appeared as a result of aerial deposition from distant sources during the boreal summer. This suggests that the *Sanguina* group may prefer conditions characteristic of the late stage of snowmelt, when the snowpack is more mature and likely contains a higher water content. qPCR targeting ASV1 (*Chloromonadinia* snow group) and ASV2 (*Sanguina* group 2) supported this seasonal shift, showing an increase in their abundance as the snow persisted through the summer months (Supplementary Fig. S9). The DNA and chemical analyses aligned in showing this seasonal progression. Neither *Sanguina* group 2 nor the *Chloromonadinia* snow groups were detected in fresh winter snow (i.e. snow less than two months old), indicating that these snow algae thrive primarily during the later stages of snowmelt in summer, when environmental conditions presumably are most favorable for their growth.

### Phylogenetic divergence and population dynamics of snow algae

**Figure 3:**
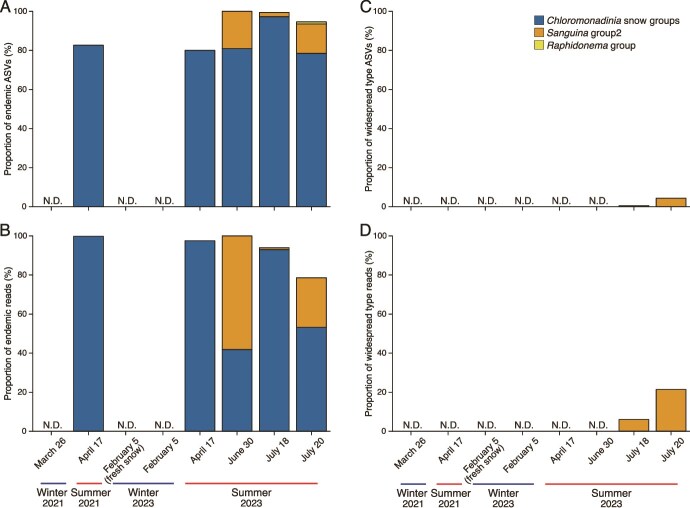
Snow algal phylogenies of endemic and widespread types in snow samples based on ITS2 sequences. Proportions of ASV sequences and the number of sequencing reads are shown. Proportions of ASV sequences (A, C) and sequencing reads (B,D) are shown for endemic (A, B) versus widespread (C, D) types.

The snow algae found on Maunakea consist of both endemic and widespread phylotypes. Here, we define phylotypes as unique ITS2 genotypes represented by DADA2-derived ASVs. ITS2 sequence analysis revealed a total of 328 ASVs, with the majority (313 ASVs: 95% of total ASVs, 90.7% of total reads) being endemic to Hawai‘i (Fig. 3, Supplementary Figs. S10–S11, Supplementary Tables S3–S7). Most of the endemic ASVs belong to the *Chloromonadinia* snow group, which is dominant from April onwards (71.4% of total reads), with *Sanguina* group 2 also being prominent (21.5% of total reads). 15 ASVs (9.3% of total reads) showed 100% sequence identity with ASVs from other regions, including Antarctica, the Arctic, and mid-latitude areas (Supplementary Table S8). These 15 ASVs include 4 from *Sanguina* group 2, 10 from *Apatococcus* and *Trebouxia* (both known lichen symbionts), and one from the Antarctic soil alga *Pseudochlorella*. Only *Sanguina* group 2 is typically found in snow and ice environments. One cosmopolitan ASV (ASV 7, 6.0% of total reads, assigned to *Sanguina* group 2 and identified as *S. nivaloides*) has been detected across both poles and mid-latitude regions [14], suggesting its widespread modern dispersal as a cosmopolitan phylotype. Presumably, the organism represented by this ASV persists in Hawai‘i only in the years when the snowpack remains into the summer, providing favorable conditions for its growth.

Phylogenetic analysis revealed that the endemic *Chloromonadinia* snow group consists of diverse lineages, divided into 10 clades (Supplementary Fig. S12). Estimated divergence times for these clades suggest that the times to most recent common ancestors (tMRCAs) were 10–130 ka (kilo annum: thousand years ago) (Supplementary Fig. S13), spanning late Pleistocene to early Holocene, with several clades predating the Last Glacial Maximum (LGM). These estimates were consistent across alternative alignments with filtered outlier lineages and rapidly evolving sites, supporting the robustness of our tMRCA results despite potential artifacts associated with ITS2 (Supplementary Fig. S14). The largest clade, Hawai‘i clade 1, consists of 171 ASVs (66% of the *Chloromonadinia* snow group) and has an estimated tMRCA of 130 ka (95% highest posterior density [HPD]: 59–225 ka). The split time between the Hawai‘i clade 1 and its closest relatives in other regions was estimated at 253 ka (95% HPD: 136–415 ka), suggesting colonization of Hawai‘i between ~253 and 130 ka. The second and third largest clades (Hawai‘i clades 2 and 3) have tMRCAs of 54 ka (95% HPD: 25–86 ka) and 41 ka (95% HPD: 14–73 ka), with split times from their nearest relatives of 126 ka (95% HPD: 60–209 ka) and 172 ka (95% HPD: 86–282 ka), respectively. These divergences suggest colonization events coinciding with or shortly after the Pohakuloa glaciation on Maunakea (MIS 6), during which snow accumulation began at ~190 ka and the summit remained ice-capped until ~120 ka [46].

**Figure 4:**
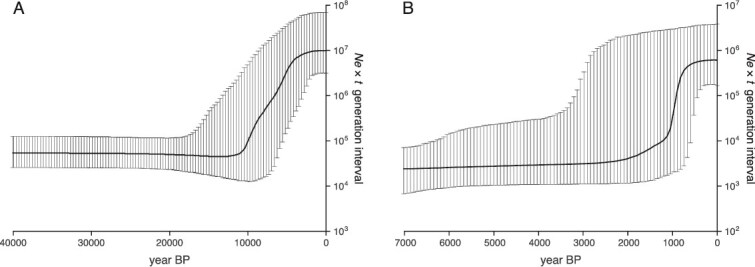
Bayesian skyline plots showing *N*_e_ × *t* for snow algae on Maunakea, where Ne is the effective population size and t is the generation interval over time. (A) Hawai‘i clade 1 of the endemic *Chloromonadinia* snow group. (B) Cosmopolitan *S. nivaloides* group. Lines represent median parameter estimates, with dashed lines representing the 95% highest posterior density interval. The *x* axis indicates years before present; the *y* axis indicates effective population size multiplied by generation interval (*N*_e_ × *t*).

Demographic history based on Bayesian skyline plot analysis indicated that the population size of the largest endemic clade 1 (Hawai‘i clade 1) increased ~10 ka (Fig. 4A), suggesting a postglaciation expansion. Hawai‘i clades 2 and 3 also showed rapid population growth around the same time, indicating similar expansion dynamics following the LGM (Supplementary Fig. S15). In contrast, the divergence times for the cosmopolitan *Sanguina* group are more recent. *S. nivaloides*, which includes cosmopolitan phylotypes, diverged ~7.4 ka, and *Sanguina aurantia* diverged ~7.8 ka (Supplementary Fig. S16). Population size increases for these groups were observed starting ~1 ka (Fig. 4B), indicating a more recent expansion for the *Sanguina* group compared to the endemic *Chloromonadinia* snow group.

## Discussion

### Conditions underlying the irregular appearance of snow algae in Hawai‘i

This study reports the occurrence of red-pigmented snow algae on the tropical Hawai‘i Island, located in the central Pacific Ocean. This finding raises important questions about the environmental conditions that enable these algae to appear and thrive in such a rare and ephemeral snow environment. The algae were detected in snow samples collected on Maunakea in 2021 and 2023. These two years exhibited contrasting snow retention durations, providing insight into the environmental conditions necessary for snow algal growth.

In 2023, an unusually long-lasting snowpack persisted into late July—an event not observed in recent decades. This prolonged snow cover was primarily due to a substantial snowfall from February 16 to 18, which deposited ~70 mm of snow-water-equivalent precipitation, followed by additional snowfalls in March and April. These successive events resulted in a thick and stable snow layer that remained well into the summer. In contrast, the 2021 snowpack was shorter-lived, but still persisted until April due to a late-season Kona Low storm on January 27. Red-pigmented algae were observed in samples from April 2021 and from June and July 2023, but not in earlier samples (Supplementary Fig. S2B). This temporal pattern suggests that snow algal blooms tend to emerge ~3 months after major snowfall events.

The timing and duration of snow retention thus appear to play a critical role in enabling snow algal proliferation, with prolonged cover providing favorable conditions for growth [8]. These observations suggest that snow algae may recolonize annually from local propagule sources, such as soil, rocks, or dormant cells within the snowpack. Their viability and activation likely depend on snow duration, and shorter snow seasons under warming conditions could restrict algal development. Understanding seasonal succession and overwintering strategies is key to predicting their responses to future climate change.

The rarity of prolonged snow cover likely explains why red snow algae have not been reported on Maunakea previously. The 2023 bloom, coinciding with La Niña-driven cooling and enhanced precipitation, underscores the sensitivity of snow algae to interannual climate variability. Snow accumulation on Maunakea is strongly influenced by the ENSO cycles. El Niño years typically result in minimal snow accumulation, as observed in 2015–2016 and 2018–2019, when snow melted within a month. In contrast, La Niña generally brings cooler, wetter conditions conducive to increased snowfall, whereas El Niño causes warmer, drier conditions with faster snowmelt [47]. The winter of 2022–2023, during the final phase of La Niña, brought cooler-than-average temperatures and substantial snowfall events, allowing the snowpack to persist for nearly eight months, representing the longest snow cover duration in recent decades. Residual snow persisting into July has been documented four times over the past 50 years (1975, 1989, 1990, and 2023) [20], with 2023 the most prolonged in recent decades. Taken together, these observations indicate that snow retention on Maunakea is tightly controlled by interannual climate variability and local radiative forcing.

One manifestation of this local forcing is enhanced direct insolation at Maunakea’s latitude—especially during the twice-annual Lāhainā Noon. Because Maunakea lies just south of the Tropic of Cancer, it experiences two Lāhainā Noon events each year (May 18 and July 24), when the sun passes directly overhead—which in 2023 coincided with the extended late spring to early summer snowpack and likely enhanced conditions for snow algal growth. The increased solar radiation during this period—especially on north-facing slopes where the snow patches are located—likely accelerates snowmelt, creating conditions favorable for algal growth [21]. Under these conditions, snowmelt infiltrates the snowpack during warm afternoons and refreezes at night, resulting in a hard snow surface by morning and a softer surface in the afternoon. By the end of summer, the snowpack reaches its highest water saturation, supporting snow algal growth.

According to a numerical model of snow algal growth, the time to reach a visible cell concentration of bloom (VCAB, defined as 5.0 × 10^5^ cells/m^2^) typically occurs from late June to September in the Northern Hemisphere [21]. Studies in the European Alps have shown that red algal blooms develop when liquid water is present throughout the snow column for more than 46 days, emphasizing the critical role of prolonged snowmelt in cold environments [48]. In Hawai‘i, snow usually disappears before the time to reach VCAB. In 2023, however, the residual snow persisted long enough for the snow algae to reach VCAB, consistent with observations of red-pigmented cells in late summer.

The growth of snow algae is governed by various factors, including melting duration, intensity, and duration of solar radiation, chemistry and nutrient availability of meltwater, and physical properties of the snow such as crystal grain size and water saturation [8]. The endemic *Chloromonadinia* snow group likely grows annually but over relatively short periods, whereas the *Sanguina* group appears to grow only during rare years when snow persists into summer (Fig. 2, Supplementary Fig. S3, Supplementary Tables S1–S2). Such sporadic and short-lived snow cover may not only constrain the duration of algal growth but also introduce episodic stressors that inhibit colonization. Comparable dynamics have been observed in snow environments influenced by volcanic activity, where ash deposition or eruptions can render the snow transiently inhospitable for algal colonization [49]. On Maunakea, the volcanic substrate—including ash and cinders—may similarly modulate microenvironmental conditions, contributing to the patchy and ephemeral distribution of algal colonization. The delayed increase of the *Sanguina* group in late July rather than May further suggests that additional factors such as snow water content and solar radiation play important roles in their growth.

Without seasonal snow patches that persist into summer, endemic snow algae (such as *Chloromonadinia* snow group) are likely able to survive the off-snow season by entering a dormant state and to resume growth when snow conditions return. However, cosmopolitan phylotypes like *Sanguina* may require longer-lasting snow conditions to complete their life cycle. Similar dormant behavior in other types of substrates, such as soils and rock surfaces, has been reported in various regions [7, 50]. The situation in Hawai‘i is particularly noteworthy due to the combination of volcanic ash and the extreme isolation of Maunakea’s cryoecosystem.

This combination of dormancy, episodic stressors, and environmental heterogeneity may not only drive the sporadic appearance of snow algae on Maunakea but also contribute to the unexpectedly high diversity observed in its snowpack. In contrast to alpine or polar sites, where red snow algal communities are typically dominated by one or a few groups, no single group was dominant in the Hawaiian snowpack (Supplementary Fig. S11). Such environmental heterogeneity may reduce the strength of competitive exclusion, facilitating the episodic establishment of multiple lineages from both local and potentially distant sources.

### Distinct dispersal processes in two major groups of snow algae

Our study reveals that snow algae on Maunakea consist of both cosmopolitan and endemic phylotypes. Cosmopolitan phylotypes, particularly those from the *Sanguina* group, have genetic connections with populations from the Antarctic, Arctic, and mid-latitude regions [5, 14, 51, 52], suggesting that long-distance dispersal between Hawai‘i and other parts of the cryosphere is ongoing. For instance, our samples included *S. nivaloides*, a species widely distributed across the global cryosphere. The continued dispersal of this species is likely facilitated by atmospheric circulation or migratory birds such as the Pacific golden plover (*Pluvialis fulva*), which travels annually between Hawai‘i and Alaska and may transport spores across vast distances [53]. However, successful colonization in Hawai‘i appears to occur only under rare climatic conditions, particularly in years with prolonged snow cover.

The *Chloromonadinia* snow group on Maunakea is predominantly endemic, with more than 95% of detected phylotypes unique to the island (Fig. 3). Geological evidence indicates that Maunakea supported an ice cap during the LGM (MIS 2) until ~15 ka, covering 70.5 km^2^ and exceeding 100 m in thickness, with summit cinder cones emerging as nunataks [46, 54]. Mean temperatures were ~7°C lower than today [46, 54], sustaining persistent cold habitats at low latitude and high elevation. Such conditions likely favored the establishment and local persistence of cold-adapted lineages, including snow algae. Therefore, the endemic phylotypes likely include relicts of past glaciations that persisted locally in Maunakea’s low-latitude, high-elevation cryoclimate.

For the largest Hawaiian *Chloromonadinia* clade, phylogenetic analyses indicate colonization between ~253 and 130 ka, overlapping the Pohakuloa glaciation (MIS 6) on Maunakea, when the summit was covered by an ice cap [46] (Supplementary Figs. S12–S13). Glacial conditions associated with the Pohakuloa glaciation may have facilitated long-distance dispersal and establishment in Hawai‘i (e.g. via atmospheric or oceanic transport). Maunakea’s high elevation (~4000 m) would have supported persistent cryospheric habitats during this glaciation. Endemic lineages appear to have persisted under these prolonged, stable cold conditions, whereas cosmopolitan counterparts are more opportunistic and tend to grow only under specific climate conditions. Following the last glacial period, the endemic *Chloromonadinia* snow group appears to have undergone substantial population growth, particularly during the warming period after the LGM. Bayesian skyline plots indicate that populations within this group began increasing around 10 ka (Fig. 4, Supplementary Fig. S15), consistent with postglacial warming and deglaciation on Maunakea. This pattern suggests adaptation to Hawai‘i’s relatively short snow seasons, with growth during sporadic but sufficiently long periods of snowpack persistence. During the off-snow season, these algae likely remain dormant in volcanic ash and other substrates, recolonizing the snow surface once conditions become favorable [8].

Despite their predominantly endemic status, some *Chloromonadinia* phylotypes exhibit wider distributions. Hawai‘i clade 1 of the *Chloromonadinia* snow group includes phylotypes found in Alaska (Gulkana Glacier on the southern coast) and Russia (Suntar-Khayata Mountains). The maximum likelihood phylogenetic tree places these phylotypes within the internal branches of Hawai‘i clade 1 (Supplementary Fig. S12), suggesting dispersal from Hawai‘i to Alaska and Russia. This implies that the *Chloromonadinia* snow group, while primarily endemic, may have contributed to dispersal events beyond the island under favorable climatic conditions in the past.

The community structure of snow algae on Maunakea is unique, shaped by both endemic and cosmopolitan phylotypes. The sporadic presence of the globally distributed *Sanguina* group on Maunakea highlights the role of long-distance dispersal, possibly via migratory birds or atmospheric circulation. However, their presence appears to depend on unusually persistent snow conditions and may result more from episodic deposition than local survival [7]. The isolated volcanic environment of Maunakea, combined with its distinctive climate and geomorphology, provides a rare context for studying cryosphere-associated dispersal dynamics. These findings underscore contrasting ecological strategies: cosmopolitan phylotypes like *Sanguina* exploit rare climatic windows, whereas endemic *Chloromonadinia* phylotypes have evolved to cope with Maunakea’s characteristically short and irregular snow seasons. These endemic lineages, likely relicts from the glacial period, have undergone unique evolutionary processes on Hawai‘i Island, contributing to the formation of distinct ecosystems that may be particularly vulnerable to the impacts of climate warming. Members of the *Chloromonadinia* snow group are obligate snow algae, known to grow exclusively on snow surfaces. On Maunakea, however, snow is both ephemeral and unreliable, with some years experiencing no snow cover at all, resulting in highly transient and unpredictable habitat availability. The persistence of this group under such conditions suggests that the Hawaiian lineage may represent a genetically distinct population uniquely adapted to survive and recolonize in a marginal cryospheric environment. Therefore, long-term genetic isolation over tens of thousands of years may result not only from limited dispersal, but also from the low probability of postdispersal survival in this harsh, discontinuous habitat. Thus, both dispersal limitation and environmental filtering likely contributed to the observed endemism.

Although cosmopolitan taxa such as *Sanguina* can arrive via long-distance dispersal, they are unlikely to persist under Hawai‘i’s characteristically short and irregular snow seasons. By contrast, endemic *Chloromonadinia* phylotypes appear better suited to these conditions, as reflected in the high proportion of endemic phylotypes in our Hawaiian samples (95% of total ASVs; 90.7% of total reads), exceeding values reported for polar and alpine regions using comparable ITS2 amplicon sequencing (mean 55.1% of total ASVs and 21.4% of total reads; Antarctica: 77.9%/64.9%, Svalbard: 49.9%/6.9%, Greenland: 21.7%/5.0%, Alaska: 70.8%/24.1%) [6]. At the same time, microbial biogeography faces the fundamental challenge of nonfalsifiability, making it difficult to determine whether observed endemism reflects true evolutionary isolation or gaps in global sampling. Broader, systematic surveys may reveal currently unsampled regions that share genetic connectivity with Hawaiian populations. Nevertheless, cosmopolitan phylotypes such as *Sanguina* have been consistently recovered from snow across polar and alpine regions, whereas the Hawaiian *Chloromonadinia* group remains undetected elsewhere despite comparable sampling efforts–a contrast supporting the interpretation that these lineages may be endemic, although undetected distributions cannot be fully excluded.

Although ITS2 has rarely been used for divergence time estimation, a few prior studies have applied it to microbial systems (e.g. 55). We evaluated its suitability by applying filtering approaches to reduce potential artifacts arising from intragenomic variation and rate heterogeneity. These included the removal of lineages with extreme evolutionary rates and the exclusion of fast-evolving sites (Supplementary Fig. S14). The resulting estimates were broadly consistent across treatments, suggesting that ITS2 can provide reasonable resolution for recent divergence events when genome-wide data are unavailable. We also considered the possible influence of intragenomic polymorphism in interpreting phylogenetic patterns (see Supplementary Results).

Our data from Hawai‘i Island show that geographically isolated, low-latitude cryospheric habitats can harbor lineages with distinct evolutionary histories. To assess the generality of these patterns and to track their sensitivity to warming, surveys should extend beyond Hawai‘i, with particular attention to conserving these rare, low-latitude cryospheric habitats.

## Supplementary Material

Supplementary_materials_wraf197

## Data Availability

The raw sequence datasets have been submitted to the DDBJ Sequence Read Archive under accession number PRJDB20005.
