# Supplementary material for: Colonization history of snow algae on Hawai‘i island
Source: ISME J. 2025 Sep 5;19(1):wraf197. doi: 10.1093/ismejo/wraf197 (PMC12449082; doi:10.1093/ismejo/wraf197)
Supplement: Supplementary_materials_wraf197 [file supplementary_materials_wraf197.pdf]

Supplemental Information

Colonization history of snow algae on Hawai‘i Island

Takahiro Segawa, Nozomu Takeuchi, Ryo Matsuzaki, Takahiro Yonezawa, Kenji Yoshikawa

This PDF file includes:

Supplemental Methods

Supplemental Figures 1–16

Supplemental Tables 1–8

Supplementary Results

Utility of ITS2 sequences for phylogenetic analysis and divergence time estimations

Our divergence time analysis based on ITS2 sequences suggests that these endemic Hawaiian lineages date back to the last glacial period. Although a few prior studies have applied ITS2 to microbial systems (e.g., 55) , it has rarely been used for divergence time estimation. This is primarily due to its high evolutionary rate and substitution-rate heterogeneity, which make accurate alignment and model fitting difficult beyond closely related taxa. In addition, morphological divergence is often limited among such taxa, thus precluding fossil-based calibration of internal nodes. As a result, ITS2 has generally been considered unsuitable for divergence time estimation, except when using tip-dating approaches that incorporate ancient DNA sequences to calibrate terminal nodes. However, such applications remain rare, and the broader suitability of ITS2 as a molecular marker for divergence time estimation has not been systematically assessed.

A further complication is the incomplete concerted evolution of ITS2 sequences, which may result in intragenomic polymorphisms. Some of the ten clades identified in this study could therefore represent paralogous copies rather than distinct lineages. Nevertheless, the emergence of multiple clades unique to Hawaiʻi is unlikely to have occurred without long-term genetic isolation of the local populations, even if our data do not allow us to determine the exact number of endemic species. These findings support the hypothesis that multiple lineages have undergone independent evolutionary trajectories in Hawaiʻi over extended timescales.

In this study, we examined two factors of uncertainty that may influence divergence time estimates based on ITS2: (1) variation in molecular evolutionary rates among

lineages, and (2) the impact of rapidly evolving nucleotide sites. To address the first, we excluded outlier lineages with exceptionally high or low substitution rates, which can bias estimates even under relaxed molecular clock models. To address the second, we filtered out the fastest-evolving sites, which can introduce homoplasy through multiple substitutions and reduce alignment accuracy. The resulting tMRCA estimates were broadly consistent across all alignment treatments (Supplementary Fig. 14), suggesting that our conclusions are robust to potential artifacts associated with ITS2-specific rate heterogeneity and alignment noise. These results support the utility of ITS2 for shallow-scale divergence time inference when ancient environmental sequences are available. However, we also recognize its inherent limitations. These include ITS2's short length, high substitution rate variability among lineages, common intragenomic polymorphisms, and its inherent unsuitability for reliable alignment and rooting across distant taxa. Accordingly, future studies incorporating genome-wide markers will be essential to refine temporal estimates and to more accurately relate divergence events to past climatic fluctuations.

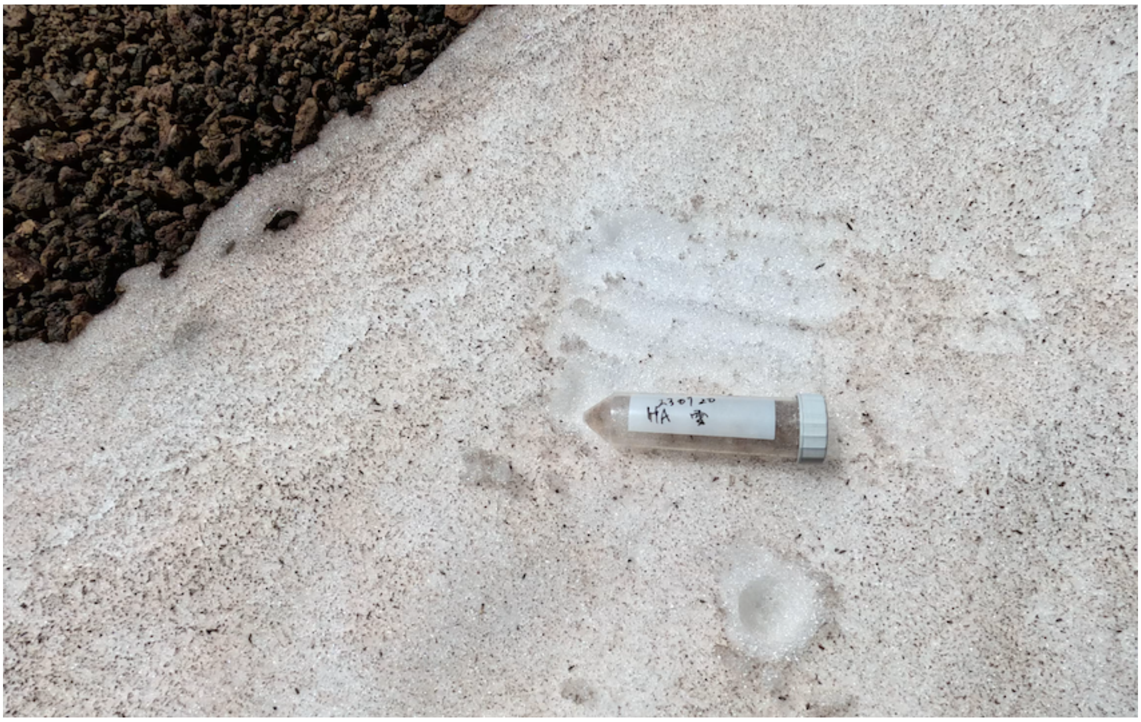

Supplementary Figure 1: Snow patch sampled on Maunakea in July 2023. Unlike the vivid red snow commonly reported in other alpine or polar regions, visible surface discoloration was minimal. Red-pigmented algal cells were detected microscopically, but the macroscopic coloration was faint, likely due to low biomass or environmental modulation of pigment production.

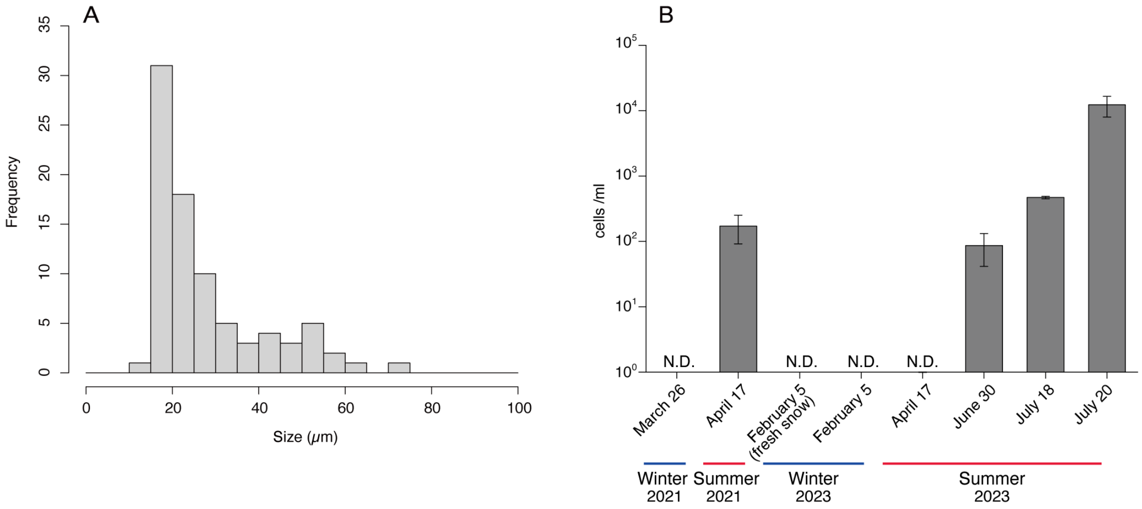

Supplementary Figure 2: Cell size distribution and seasonal variation in cell numbers of red snow algae. (A) Histogram of cell sizes for red snow algae. The frequency of cells for each size range is shown. (B) Seasonal change of snow algae cell numbers on Maunakea estimated by direct microscopic count.

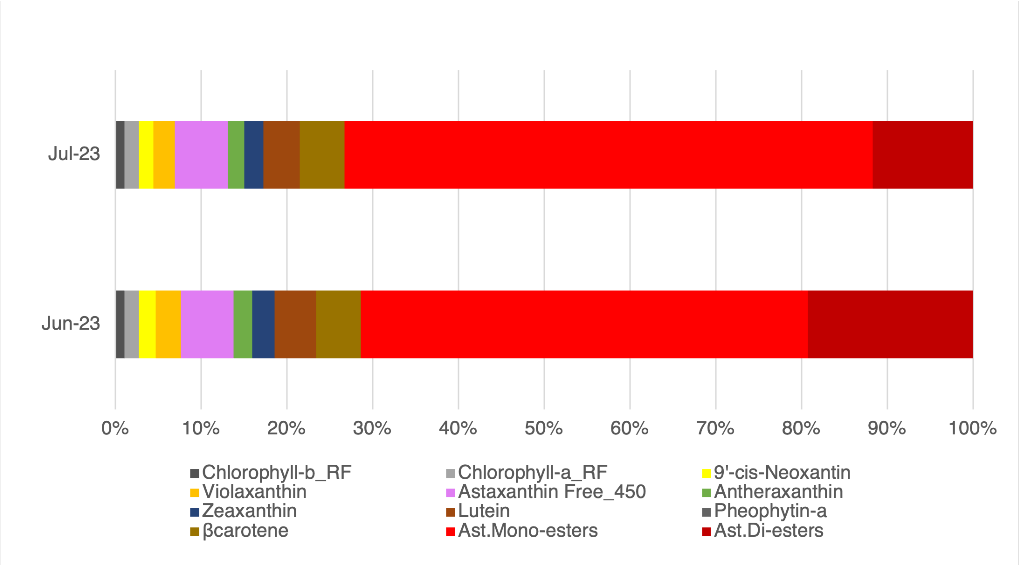

Supplementary Figure 3: HPLC chromatograms of pigment types of red snow samples in this study (detection wavelength: 450 nm).

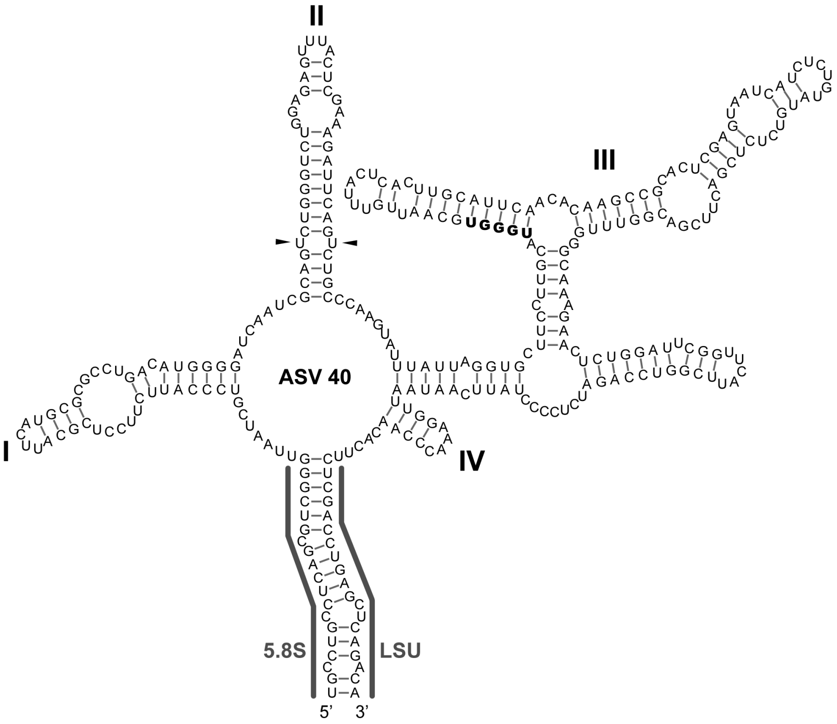

Supplementary Figure 4: Predicted secondary structure of the nuclear rDNA ITS2 transcript of *Chloromonadinia* snow group 13 (Unique sequence no. ASV 40). The 3′ end of the 5.8S rRNA and the 5′ end of the large subunit of rRNA (LSU rRNA) are indicated by dark lines. Note the U-U mismatch in helix II (arrowheads) and the YGGY motif (UGGGU) on the 5′ side of helix III (bold).

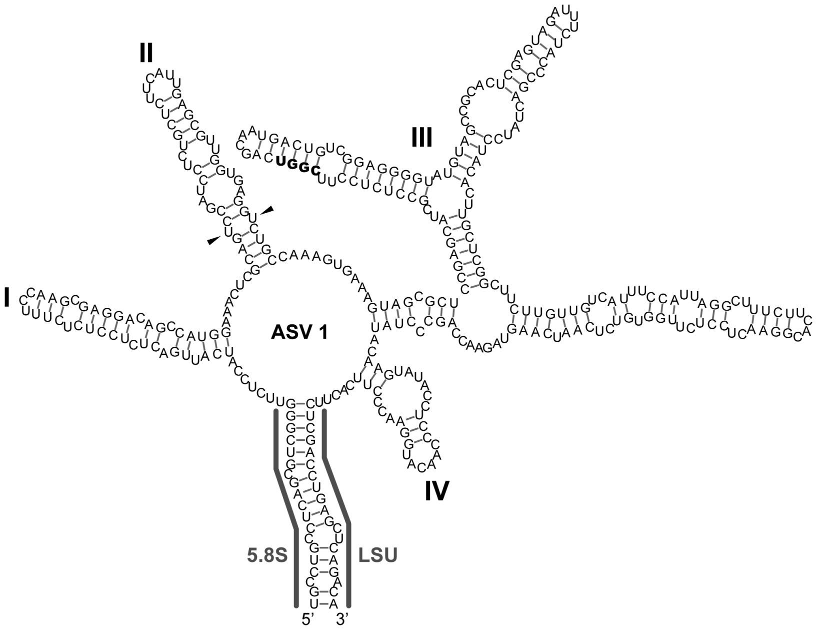

Supplementary Figure 5: Predicted secondary structure of the nuclear rDNA ITS2 transcript of *Chloromonadinia* snow group 28 (Unique sequence no. ASV 1). The 3′ end of the 5.8S rRNA and the 5′ end of the LSU rRNA are indicated by dark lines. Note the U-U mismatch in helix II (arrowheads) and the YGGY motif (CGGU) on the 5′ side of helix III (bold).

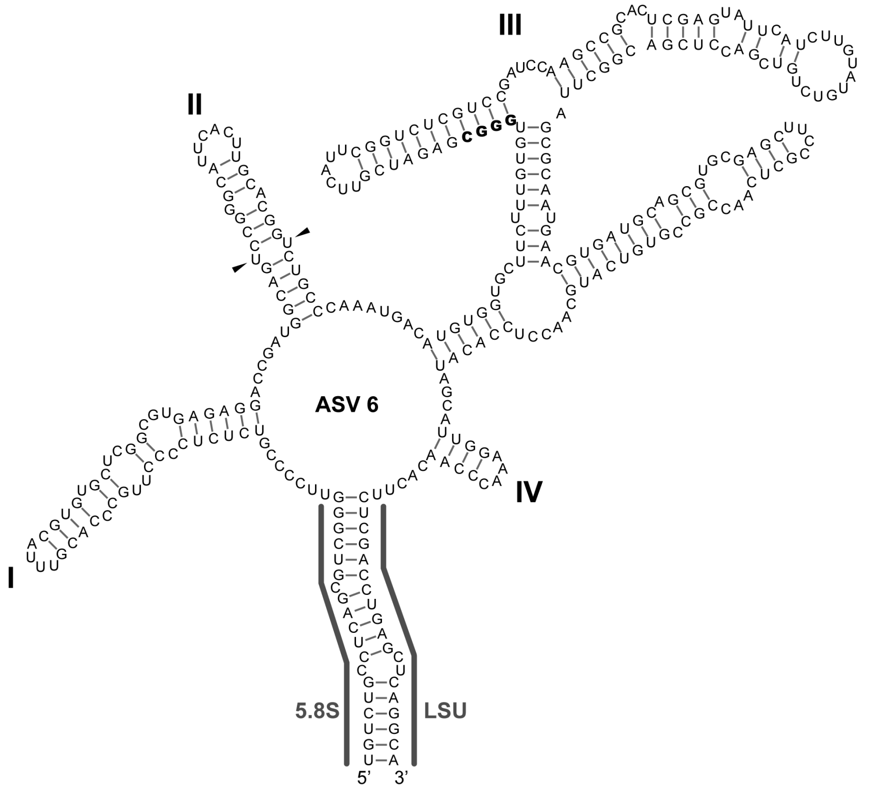

Supplementary Figure 6: Predicted secondary structure of the nuclear rDNA ITS2 transcript of *Chloromonadinia* snow group 30 (Unique sequence no. ASV 6). The 3′ end of the 5.8S rRNA and the 5′ end of the LSU rRNA are indicated by dark lines. Note the U-U mismatch in helix II (arrowheads) and the YGGY motif (GGGC) on the 5′ side of helix III (bold).

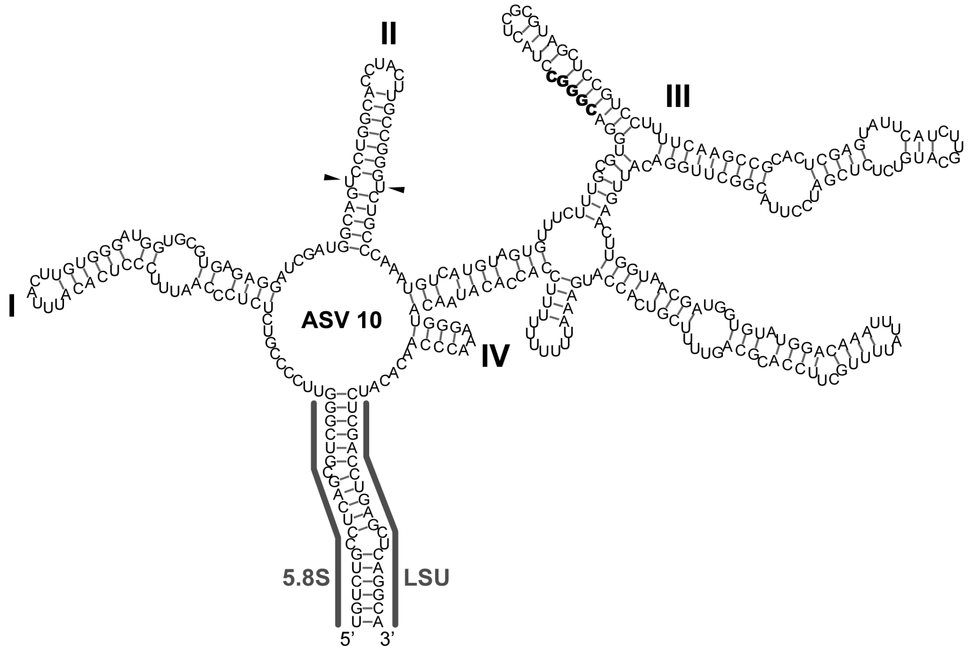

Supplementary Figure 7: Predicted secondary structure of the nuclear rDNA ITS2 transcript of *Chloromonadinia* snow group 32 (Unique sequence no. ASV 10). The 3′ end of the 5.8S rRNA and the 5′ end of the LSU rRNA are indicated by dark lines. Note the U-U mismatch in helix II (arrowheads) and the YGGY motif (CGGGC) on the 5′ side of helix III (bold).

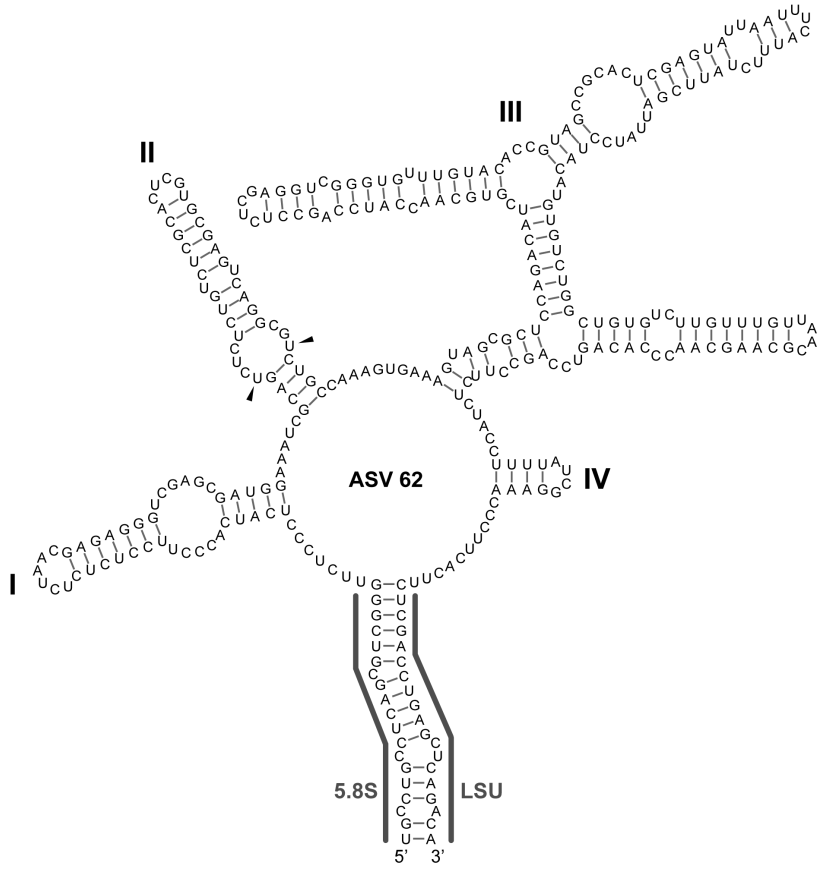

Supplementary Figure 8: Predicted secondary structure of the nuclear rDNA ITS2 transcript of *Chloromonadinia* snow group 34 (Unique sequence no. ASV 62). The 3′ end of the 5.8S rRNA and the 5′ end of the LSU rRNA are indicated by dark lines. Note the U-U mismatch in helix II (arrowheads).

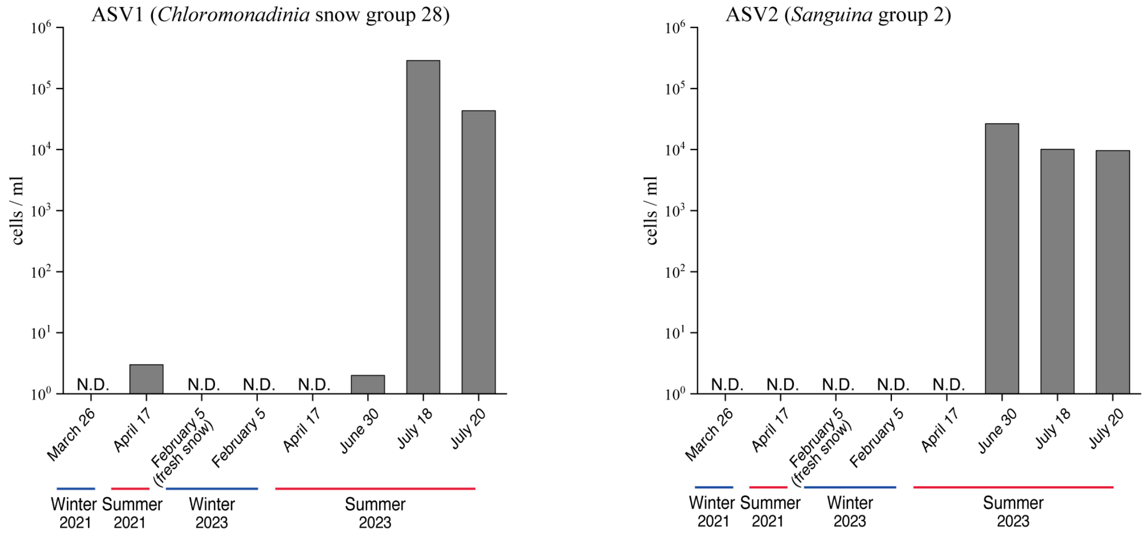

Supplementary Figure 9: Seasonal variation in the copy numbers of ASV1 (*Chloromonadinia* snow group) and ASV2 (*Sanguina* group 2) in snow samples, measured by qPCR.

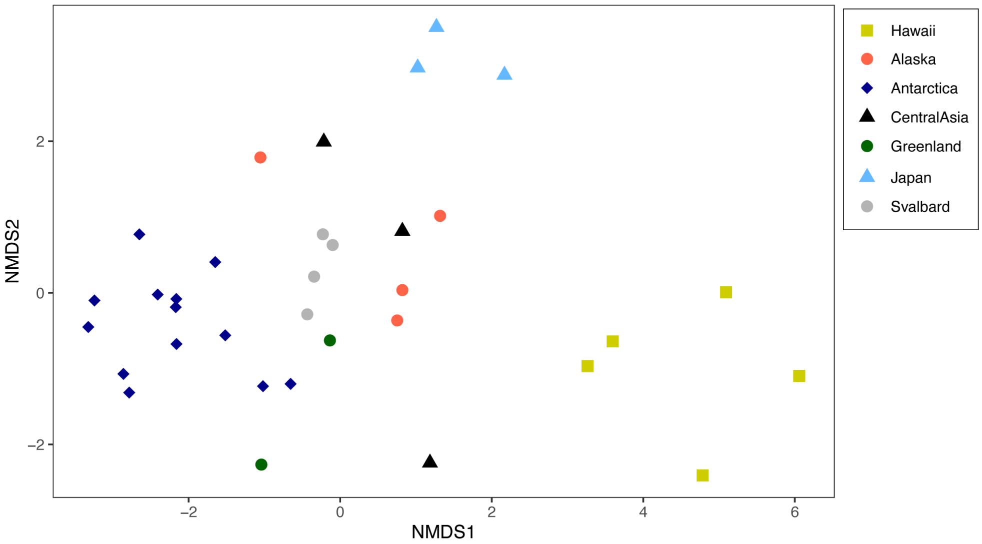

Supplementary Figure 10: Dissimilarities among algal communities based on nonmetric multidimensional scaling (NMDS). The NMDS plot was generated using the abundance matrix of unique ITS2 sequences. Each point represents an algal community from snow samples obtained from various regions.

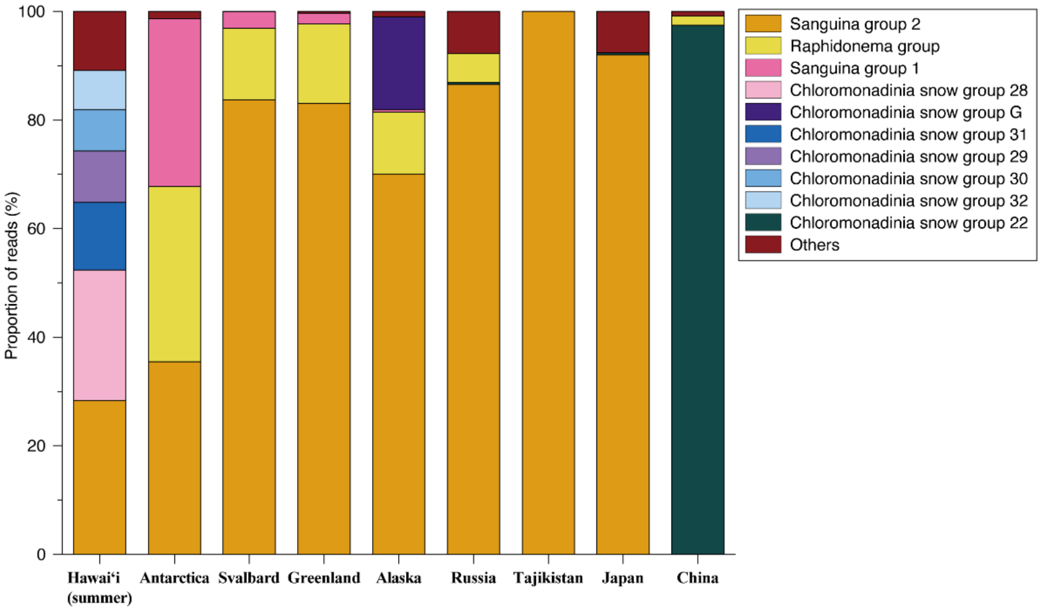

Supplementary Figure 11: Algal taxonomic composition of reads in snow samples based on ITS2 sequences. The bar chart presents the average community composition for each region, categorized into ten major ITS2 groups plus low-abundance groups (grouped as "Others"). " Hawai‘i (summer)" represents the average composition from samples collected between April and July, 2023.

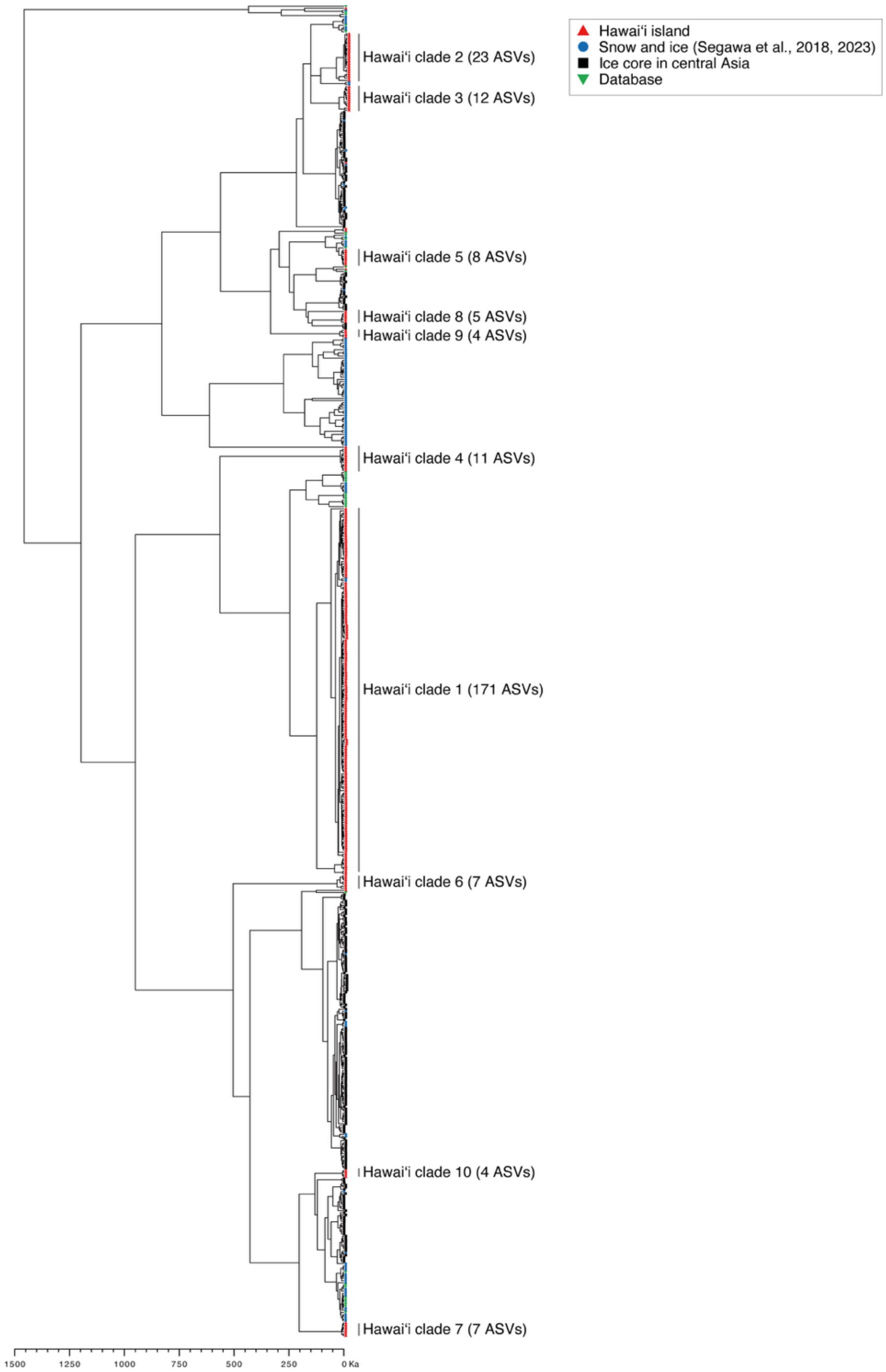

Supplementary Figure 12: Tip-calibrated coalescence times based on ITS2 sequences within the *Chloromonadinia* snow group analyzed by BEAST v. 1.10.4. Branch labels indicate the median estimated divergence time and blue bars represent the 95% highest posterior densities. Each point represents an ASV from: snow and ice samples from Hawaiʻi Island; snow and ice samples from the Antarctic, Arctic, and mid-latitude regions (sequences from Segawa et al., 2018, 2023); ice core samples from Central Asia (sequences from Segawa et al., 2023); and the NCBI non-redundant Nucleotide (nt) database.

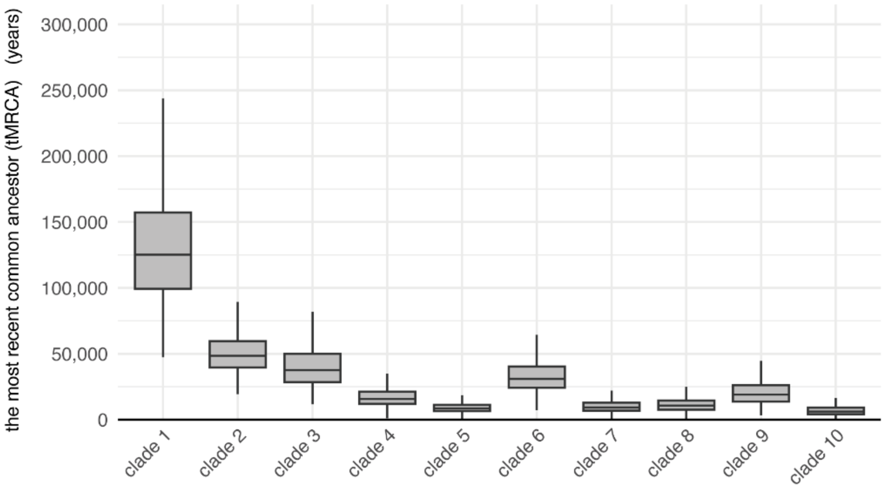

Supplementary Figure 13: Time to most recent common ancestor (tMRCA) analysis for all Hawai‘i clades (1-10) in the *Chloromonadinia* snow group. Divergence times estimated using BEAST. Each boxplot shows the posterior distribution of tMRCA for each clade: boxes represent the interquartile range (IQR; 25th to 75th percentiles), horizontal lines denote the medians, and whiskers extend to 1.5 × IQR.

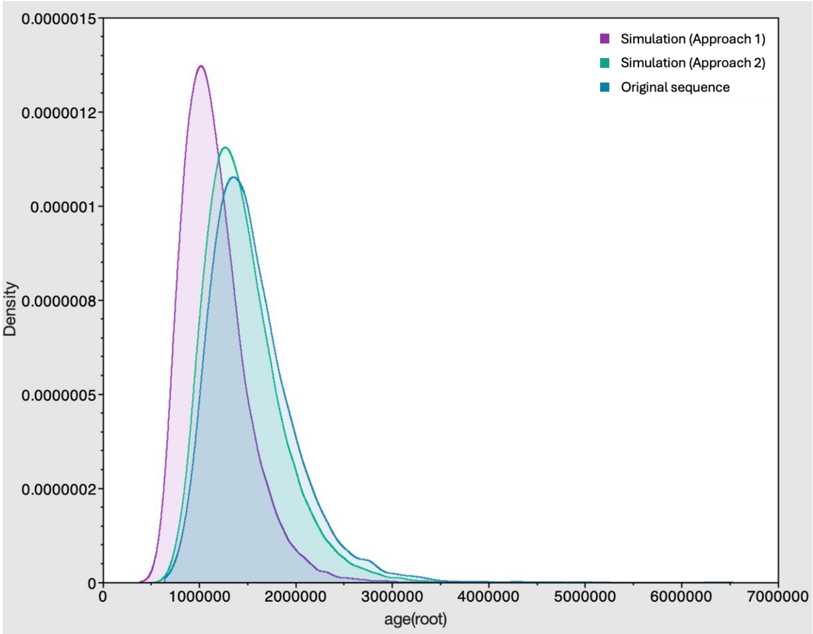

Supplementary Figure 14: Evaluation of the suitability of the ITS2 region for divergence time estimation.

We tested two approaches to assess the robustness of divergence time estimates based on ITS2 sequences: **Approach 1**: Sites with the highest substitution rates were removed. A discrete gamma distribution was used to model site rate heterogeneity, and the top 10% fastest-evolving sites were excluded from the alignment. **Approach 2**: Lineages with extremely high or low substitution rates were excluded. Root-to-tip branch lengths were calculated for all sequences, and those with Z-scores exceeding ±2 standard deviations from the mean were removed.

In order to account for factors that can significantly influence divergence time estimation—such as heterogeneity in molecular evolutionary rates among lineages and the effects of multiple substitutions—we estimated divergence times using alignments from which such sites or lineages had been removed. Analyses were performed under a relaxed molecular clock within a hierarchical Bayesian framework.

The posterior distributions of the time to the tMRCA, derived from these modified alignments, closely resembled those obtained from the original dataset. This result suggests that divergence time estimates based on ITS2 sequences are robust to rate heterogeneity across both sites and lineages, and supports the utility of ITS2 as a genetic marker for divergence time inference.

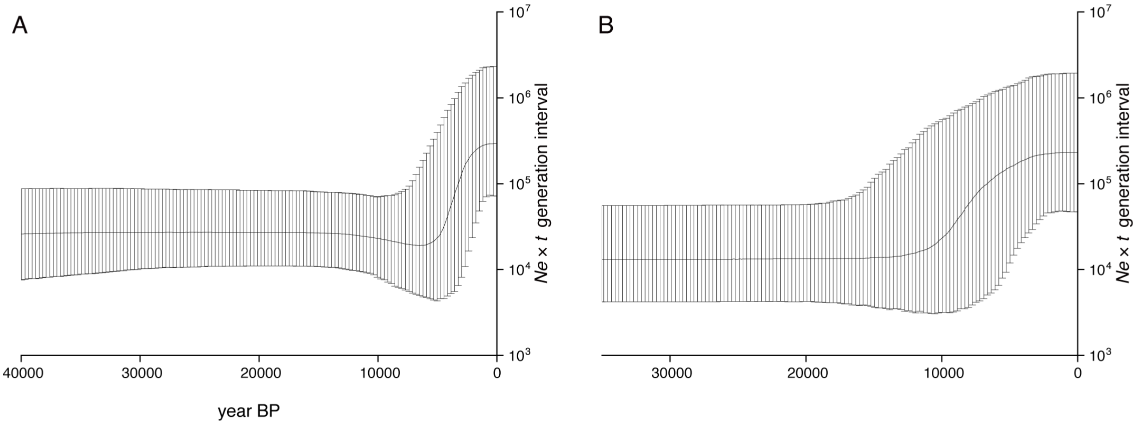

Supplementary Figure 15: Bayesian skyline plots showing $N_e \times t$ for snow algae on Maunakea, where Ne is effective population size and t is generation interval over time. **(A)** Hawai‘i clade 2 of the endemic *Chloromonadinia* snow group. **(B)** Hawai‘i clade 3 of the endemic *Chloromonadinia* snow group. Dashed lines represent the 95% highest posterior density interval. The *x* axis indicates years before present; the *y* axis indicates effective population size multiplied by generation interval ($N_e \times t$).

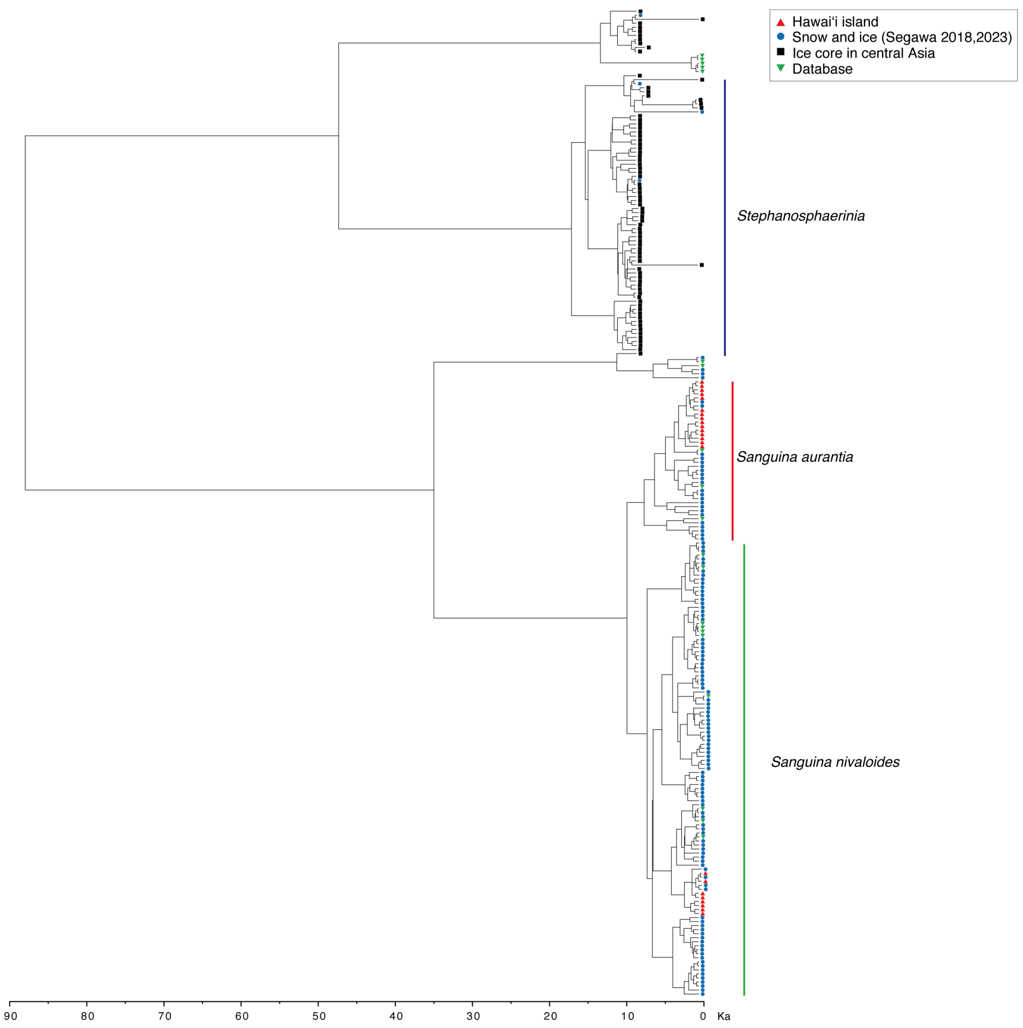

Supplementary Figure 16: Tip-calibrated coalescence times based on ITS2 sequences within the *Sanguina* group analyzed by BEAST v. 1.10.4. Branch labels indicate the median estimated divergence time and blue bars represent the 95% highest posterior densities. Each point represents an ASV from: snow and ice samples Hawai'i Island; snow and ice from the Antarctic, Arctic, and mid-latitude regions (sequences from Segawa et al., 2018, 2023); ice core samples from central Asia (sequences from Segawa et al., 2023); and the NCBI non-redundant Nucleotide (nt) database.

Supplementary Table 1: Ratio for each taxonomy group at Maunakea on Hawai'i Island.

		2021/3/26	2021/4/17	2023/2/5 (fresh snow)	2023/2/5	2023/4/17	2023/6/30	2023/7/18	2023/7/20
Chlorophyceae	Sanguina group 2	0	0	0	0	0	58.2	7.0	46.7
	Chloromonadinia snow group 11	0	14.5	0	0	0	0	0	0.8
	Chloromonadinia snow group 13	0	0.7	0	0	0	0	2.8	6.2
	Chloromonadinia snow group 28	0	0	0	0	0	0	84.4	34.6
	Chloromonadinia snow group 29	0	38.2	0	0	7.6	0.02	0.2	1.2
	Chloromonadinia snow group 30	0	0	0	0	89.3	0	0	0.2
	Chloromonadinia snow group 31	0	46.4	0	0	0.0	0.01	0.8	7.5
	Chloromonadinia snow group 32	0	0	0	0	0.5	28.6	0.1	0
	Chloromonadinia snow group 33	0	0	0	0	0	9.3	0	0.1
	Chloromonadinia snow group 34	0	0	0	0	0	2.8	2.3	0.1
	Chloromonadinia snow group 35	0	0.01	0	0	0	0	2.1	1.8
	Chloromonadinia snow group 36	0	0	0	0	0	0.8	0	0
	Chloromonadinia snow group 37	0	0	0	0	0	0	0.03	0.6
	Chloromonadinia snow group 38	0	0	0	0	0	0.2	0	0
	Chloromonadinia snow group 39	0	0	0	0	0	0.1	0	0
	Chloromonadinia snow group 40	0	0	0	0	0.1	0	0.2	0.003
	Chloromonadinia snow group 41	0	0	0	0	0	0	0.2	0.05
	Chloromonadinia snow group 42	0	0	0	0	0	0	0	0.003
	Chloromonadinia snow group 43	0	0.003	0	0	0	0	0	0
	Chloromonadinia snow group 44	0	0	0	0	0	0	0	0.002
Trebouxiophyceae	Apatococcus group	0	0	10.2	20.0	0	0	0	0
	Dictyochloropsis group	1.4	0	1.8	3.2	0	0	0	0
	Diplosphaera group	0	0	0.2	0	0	0	0	0
	Myrmecia group	0	0	0.4	2.5	0	0	0	0
	Neocystis group	0	0	0.05	0	0	0	0	0
	Raphidonema group	0	0	0	0	0	0	0	0.04
	Pseudochlorella group	1.2	0	0.1	0	0	0	0	0
	Stichococcus group	4.3	0.02	0.1	0.2	0	0	0	0
	Trebouxia CBC group 1	0	0	0.8	0	0	0	0	0
	Trebouxia CBC group 3	0	0	6.1	13.8	0	0	0	0
	Trebouxia CBC group 4	1.4	0	0	0	0	0	0	0
	Trebouxia CBC group 5	9.7	0.03	0	0	0	0	0	0
	Trebouxia CBC group 6	11.5	0	0	0	0	0	0	0
	Trebouxia CBC group 7	24.5	0	0.2	0.3	0	0	0	0
	Trebouxia CBC group 8	12.8	0.02	0.9	0.4	0	0	0	0
	Trebouxia CBC group 9	28.3	0.2	78.9	59.1	2.4	0	0	0
	Trebouxia CBC group 10	4.9	0	0	0	0	0	0	0.04
	Trebouxia CBC group 11	0	0	0	0.3	0	0	0	0
	Trebouxia CBC group 12	0	0	0.2	0.2	0	0	0	0
	Trebouxia CBC group 13	0	0	0	0	0.1	0	0	0

Supplementary Table 2: Number of ASVs for each taxonomy group at Maunakea on Hawai‘i Island.

		2021/3/26	2021/4/17	2023/2/5 (fresh snow)	2023/2/5	2023/4/17	2023/6/30	2023/7/18	2023/7/20
Chlorophyceae	Sanguina group 2	0	0	0	0	0	9	5	18
	Chloromonadinia snow group 11	0	1	0	0	0	0	0	1
	Chloromonadinia snow group 13	0	2	0	0	0	0	6	7
	Chloromonadinia snow group 28	0	0	0	0	0	0	151	43
	Chloromonadinia snow group 29	0	7	0	0	1	1	1	1
	Chloromonadinia snow group 30	0	0	0	0	12	0	0	1
	Chloromonadinia snow group 31	0	7	0	0	1	1	3	4
	Chloromonadinia snow group 32	0	0	0	0	1	23	1	0
	Chloromonadinia snow group 33	0	0	0	0	0	4	0	1
	Chloromonadinia snow group 34	0	0	0	0	0	6	7	1
	Chloromonadinia snow group 35	0	1	0	0	0	0	5	5
	Chloromonadinia snow group 36	0	0	0	0	0	1	0	0
	Chloromonadinia snow group 37	0	0	0	0	0	0	1	4
	Chloromonadinia snow group 38	0	0	0	0	0	1	0	0
	Chloromonadinia snow group 39	0	0	0	0	0	1	0	0
	Chloromonadinia snow group 40	0	0	0	0	1	0	1	1
	Chloromonadinia snow group 41	0	0	0	0	0	0	3	1
	Chloromonadinia snow group 42	0	0	0	0	0	0	0	1
	Chloromonadinia snow group 43	0	1	0	0	0	0	0	0
	Chloromonadinia snow group 44	0	0	0	0	0	0	0	2
Trebouxiophyceae	Apatococcus group	0	0	2	2	0	0	0	0
	Dictyochloropsis group	1	0	1	1	0	0	0	0
	Diplosphaera group	0	0	1	0	0	0	0	0
	Myrmecia group	0	0	1	1	0	0	0	0
	Neocystis group	0	0	1	0	0	0	0	0
	Raphidonema group	0	0	0	0	0	0	0	1
	Pseudochlorella group	1	0	1	0	0	0	0	0
	Stichococcus group	1	1	1	1	0	0	0	0
	Trebouxia CBC group 1	0	0	2	0	0	0	0	0
	Trebouxia CBC group 3	0	0	1	1	0	0	0	0
	Trebouxia CBC group 4	1	0	0	0	0	0	0	0
	Trebouxia CBC group 5	1	1	0	0	0	0	0	0
	Trebouxia CBC group 6	1	0	0	0	0	0	0	0
	Trebouxia CBC group 7	3	0	2	1	0	0	0	0
	Trebouxia CBC group 8	2	1	3	1	0	0	0	0
	Trebouxia CBC group 9	2	1	12	8	3	0	0	0
	Trebouxia CBC group 10	1	0	0	0	0	0	0	1
	Trebouxia CBC group 11	0	0	0	1	0	0	0	0
	Trebouxia CBC group 12	0	0	1	1	0	0	0	0
	Trebouxia CBC group 13	0	0	0	0	1	0	0	0
	Total	14	23	29	18	20	47	184	93

Supplementary Table 3: Proportion of read sequences for each taxonomy group in the various regions

		Hawai'i (summer)	Antarctica	Svalbard	Greenland	Alaska	Russia	Tajikistan	Japan	China	Reference
Chlorophyceae	Sanguina group 1	-	30.92	3.04	1.98	0.44	0.01	-	-	-	Segawa et al. 2018
	Sanguina group 2	28.32	35.49	83.67	83.00	70.03	86.56	100	91.99	-	Segawa et al. 2018
	Chloromonadinia snow group 1	-	-	-	-	-	-	-	0.08	-	Segawa et al. 2023
	Chloromonadinia snow group 2	-	-	-	-	-	0.04	-	-	-	Segawa et al. 2023
	Chloromonadinia snow group 3	-	-	-	-	-	-	-	-	0.06	Segawa et al. 2023
	Chloromonadinia snow group 4	0.04	-	-	-	-	0.22	-	0.01	-	Segawa et al. 2023
	Chloromonadinia snow group 6	-	-	-	-	-	1.21	-	-	-	Segawa et al. 2023
	Chloromonadinia snow group 7	-	-	-	-	-	0.78	-	-	-	Segawa et al. 2023
	Chloromonadinia snow group 9	-	-	-	-	-	-	-	0.70	-	Segawa et al. 2023
	Chloromonadinia snow group 10	-	-	-	-	-	1.28	-	-	0.15	Segawa et al. 2023
	Chloromonadinia snow group 11	3.42	-	-	-	-	-	-	-	0.17	Segawa et al. 2023
	Chloromonadinia snow group 12	-	-	-	-	-	0.01	-	-	-	Segawa et al. 2023
	Chloromonadinia snow group 13	2.29	-	-	-	-	0.02	-	0.004	-	Segawa et al. 2023
	Chloromonadinia snow group 18	-	-	-	-	-	0.10	-	-	-	Segawa et al. 2023
	Chloromonadinia snow group 19	-	-	-	-	-	0.87	-	-	-	Segawa et al. 2023
	Chloromonadinia snow group 20	-	-	-	-	-	-	-	0.02	-	Segawa et al. 2023
	Chloromonadinia snow group 21	-	-	-	-	-	0.03	-	-	-	Segawa et al. 2023
	Chloromonadinia snow group 22	-	-	-	-	-	0.35	-	0.37	97.44	Segawa et al. 2023
	Chloromonadinia snow group 23	-	-	-	-	-	-	-	0.61	-	Segawa et al. 2023
	Chloromonadinia snow group 24	-	-	-	-	-	-	-	6.21	-	Segawa et al. 2023
	Chloromonadinia snow group 25	-	-	-	-	-	0.01	-	-	-	Segawa et al. 2023
	Chloromonadinia snow group 26	-	-	-	-	0.15	1.19	-	-	-	Segawa et al. 2023
	Chloromonadinia snow group 27	-	-	-	-	0.04	1.98	-	-	-	Segawa et al. 2023
	Chloromonadinia snow group 28	24.05	-	-	-	-	-	-	-	-	This study
	Chloromonadinia snow group 29	9.46	-	-	-	-	-	-	-	-	This study
	Chloromonadinia snow group 30	7.60	-	-	-	-	-	-	-	-	This study
	Chloromonadinia snow group 31	12.46	-	-	-	-	-	-	-	-	This study
	Chloromonadinia snow group 32	7.23	-	-	-	-	-	-	-	-	This study
	Chloromonadinia snow group 33	2.36	-	-	-	-	-	-	-	-	This study
	Chloromonadinia snow group 34	1.15	-	-	-	-	-	-	-	-	This study
	Chloromonadinia snow group 35	0.85	-	-	-	-	-	-	-	-	This study
	Chloromonadinia snow group 36	0.21	-	-	-	-	-	-	-	-	This study
	Chloromonadinia snow group 37	0.16	-	-	-	-	-	-	-	-	This study
	Chloromonadinia snow group 38	0.04	-	-	-	-	-	-	-	-	This study
	Chloromonadinia snow group 39	0.03	-	-	-	-	-	-	-	-	This study
	Chloromonadinia snow group 40	0.04	-	-	-	-	-	-	-	-	This study
	Chloromonadinia snow group 41	0.001	-	-	-	-	-	-	-	-	This study
	Chloromonadinia snow group 42	0.001	-	-	-	-	-	-	-	-	This study
	Chloromonadinia snow group 44	0.001	-	-	-	-	-	-	-	-	This study
	Chloromonadinia snow group A	-	0.10	0.05	0.002	0.58	-	-	-	-	Segawa et al. 2018
	Chloromonadinia snow group B	-	0.18	-	0.34	0.21	-	-	-	-	Segawa et al. 2018
	Chloromonadinia snow group C	-	0.58	-	-	-	-	-	-	-	Segawa et al. 2018
	Chloromonadinia snow group D	-	-	0.002	-	0.03	-	-	-	-	Segawa et al. 2018
	Chloromonadinia snow group G	-	-	0.0005	-	17.09	-	-	-	-	Segawa et al. 2018
	Moewusinia group A	-	0.36	0.004	-	-	-	-	-	-	Segawa et al. 2018
	Moewusinia group B	-	0.001	-	-	-	-	-	-	-	Segawa et al. 2018
	Monadinia group	-	-	-	-	-	-	-	-	0.05	Segawa et al. 2023
	Stephanosphaerinia group	-	0.09	-	-	-	-	-	-	-	Segawa et al. 2018
	Stephanosphaerinia group 1	-	-	-	-	-	-	-	-	0.16	Segawa et al. 2023
	unnamed Chlorophyceae group A	-	0.004	-	-	-	-	-	-	-	Segawa et al. 2018
Trebouxiophyceae	Asterochloris group	-	-	0.01	-	0.0004	-	-	-	-	Segawa et al. 2018
	Chlorella group 1	-	-	-	-	-	-	-	-	0.01	Segawa et al. 2023
	Chlorella group 2	-	-	-	-	-	-	-	-	0.18	Segawa et al. 2023
	Chlorella group	-	0.005	-	-	-	-	-	-	-	Segawa et al. 2018
	Chloroidium group A	-	0.002	-	-	-	-	-	-	-	Segawa et al. 2018
	Chloroidium group B	-	0.01	-	-	-	-	-	-	-	Segawa et al. 2018
	Chloroidium group C	-	-	-	-	0.001	-	-	-	-	Segawa et al. 2018
	Elliptochloris group	-	-	-	-	-	0.01	-	-	-	Segawa et al. 2023
	Raphidonema group	0.01	32.21	13.23	14.67	11.43	5.31	-	-	1.67	Segawa et al. 2018
	Stichococcus group	0.004	-	-	-	-	-	-	-	-	This study
	Trebouxia group 1	-	-	-	-	-	0.02	-	-	-	Segawa et al. 2023
	Trebouxia group 2	-	-	-	-	-	-	-	-	0.08	Segawa et al. 2023
	Trebouxia group 3	-	-	-	-	-	-	-	-	0.02	Segawa et al. 2023
	Trebouxia group 5	0.01	-	-	-	-	-	-	-	-	This study
	Trebouxia group 8	0.004	-	-	-	-	-	-	-	-	This study
	Trebouxia group 9	0.24	-	-	-	-	-	-	-	-	This study
	Trebouxia group 10	0.01	-	-	-	-	-	-	-	-	This study
	Trebouxia group 13	0.01	-	-	-	-	-	-	-	-	This study
	Trebouxia group A	-	0.01	-	-	-	-	-	-	-	Segawa et al. 2018
	Trebouxia group B	-	0.04	0.0005	0.003	0.003	-	-	-	-	Segawa et al. 2018
	Pseudostichococcus group	-	0.001	-	-	-	-	-	-	-	Segawa et al. 2018

* Hawaii (summer) summer represents the average from April to July in 2023.

Supplementary Table 4: Proportion of read sequences detected in Hawai'i from the Antarctic or Arctic, and Mid-Lati for each taxonomy group.

	2021/3/26	2021/4/17	2023/2/5 (fresh snow)	2023/2/5	2023/4/17	2023/6/30	2023/7/18	2023/7/20
Sanguina group 2	0	0	0	0	0	0	6.00	21.40
Pseudochlorella group	0	0	0.07	0	0	0	0	0
Apatococcus group	0	0	3.05	5.34	0	0	0	0
Trebouxia CBC group 1	0	0	0.79	0	0	0	0	0
Trebouxia CBC group 5	9.72	0	0	0	0	0	0	0
Trebouxia CBC group 6	11.54	0	0	0	0	0	0	0
Trebouxia CBC group 7	13.97	0	0.14	0	0	0	0	0
Trebouxia CBC group 8	1.21	0.02	0.65	0	0	0	0	0
Trebouxia CBC group 9	0	0.16	43.29	1.92	0.18	0	0	0
Trebouxia CBC group 13	0	0	0	0	0.11	0	0	0
	36.44	0.18	47.99	7.26	0.29	0	6.00	21.40

Supplementary Table 5: Proportion of read sequences of endemic ASVs detected in Hawai'i for each taxonomy group

	2021/3/26	2021/4/17	2023/2/5 (fresh snow)	2023/2/5	2023/4/17	2023/6/30	2023/7/18	2023/7/20
Sanguina group 2	0	0	0	0	0	58.17	0.96	25.29
Raphidonema group	0	0	0	0	0	0	0	0.04
Pseudochlorella group	1.21	0	0	0	0	0	0	0
Chloromonadinia snow group 11	0	14.46	0	0	0	0	0	0.80
Chloromonadinia snow group 13	0	0.68	0	0	0	0	2.76	6.20
Chloromonadinia snow group 28	0	0	0	0	0	0	84.42	34.65
Chloromonadinia snow group 29	0	38.17	0	0	7.58	0.02	0.20	1.19
Chloromonadinia snow group 30	0	0	0	0	89.29	0	0	0.20
Chloromonadinia snow group 31	0	46.45	0	0	0.05	0.01	0.81	7.53
Chloromonadinia snow group 32	0	0	0	0	0.47	28.56	0.08	0
Chloromonadinia snow group 33	0	0	0	0	0	9.28	0	0.10
Chloromonadinia snow group 34	0	0	0	0	0	2.81	2.31	0.13
Chloromonadinia snow group 35	0	0.01	0	0	0	0	2.11	1.79
Chloromonadinia snow group 36	0	0	0	0	0	0.85	0	0
Chloromonadinia snow group 37	0	0	0	0	0	0	0.03	0.59
Chloromonadinia snow group 38	0	0	0	0	0	0.17	0	0
Chloromonadinia snow group 39	0	0	0	0	0	0.13	0	0
Chloromonadinia snow group 40	0	0	0	0	0.14	0	0.16	0.003
Chloromonadinia snow group 41	0	0	0	0	0	0	0.18	0.05
Chloromonadinia snow group 42	0	0	0	0	0	0	0	0.003
Chloromonadinia snow group 43	0	0.003	0	0	0	0	0	0
Chloromonadinia snow group 44	0	0	0	0	0	0	0	0.002
Apatococcus group	0	0	7.18	14.61	0	0	0	0
Dictyochloropsis group	1.42	0	1.80	3.24	0	0	0	0
Diplosphaera group	0	0	0.18	0	0	0	0	0
Myrmecia group	0	0	0.43	2.49	0	0	0	0
Neocystis group	0	0	0.05	0	0	0	0	0
Stichococcus group	4.25	0.02	0.15	0.20	0	0	0	0
Trebouxia CBC group 3	0	0	6.07	13.83	0	0	0	0
Trebouxia CBC group 4	1.42	0	0	0	0	0	0	0
Trebouxia CBC group 5	0	0.03	0	0	0	0	0	0
Trebouxia CBC group 7	10.53	0	0.10	0.33	0	0	0	0
Trebouxia CBC group 8	11.54	0	0.24	0.37	0	0	0	0
Trebouxia CBC group 9	28.34	0	35.64	57.15	2.18	0	0	0
Trebouxia CBC group 10	4.86	0	0	0	0	0	0	0.04
Trebouxia CBC group 11	0	0	0	0.31	0	0	0	0
Trebouxia CBC group 12	0	0	0.17	0.21	0	0	0	0
Total	63.56	99.82	52.01	92.74	99.71	100.00	94.00	78.60

Supplementary Table 6: Number of ASVs detected in Hawai‘i from the Antarctic or Arctic, and Mid-Latitudes for each taxonomy group.

	2021/3/26	2021/4/17	2023/2/5 (fresh snow)	2023/2/5	2023/4/17	2023/6/30	2023/7/18	2023/7/20
Sanguina group 2	0	0	0	0	0	0	1	4
Pseudochlorella group	0	0	1	0	0	0	0	0
Apatococcus group	0	0	1	1	0	0	0	0
Trebouxia CBC group 1	0	0	2	0	0	0	0	0
Trebouxia CBC group 5	1	0	0	0	0	0	0	0
Trebouxia CBC group 6	1	0	0	0	0	0	0	0
Trebouxia CBC group 7	1	0	1	0	0	0	0	0
Trebouxia CBC group 8	1	1	1	0	0	0	0	0
Trebouxia CBC group 9	0	1	1	1	1	0	0	0
Trebouxia CBC group 13	0	0	0	0	1	0	0	0
Total	4	2	7	2	2	0	1	4

Supplementary Table 7: Number of endemic ASVs detected in Hawai‘i for each taxonomy group.

taxonomy	2021/3/26	2021/4/17	2023/2/5 (fresh snow)	2023/2/5	2023/4/17	2023/6/30	2023/7/18	2023/7/20
Sanguina group 2	0	0	0	0	0	9	4	14
Chloromonadinia snow group 11	0	1	0	0	0	0	0	1
Chloromonadinia snow group 13	0	2	0	0	0	0	6	7
Chloromonadinia snow group 28	0	0	0	0	0	0	151	43
Chloromonadinia snow group 29	0	7	0	0	1	1	1	1
Chloromonadinia snow group 30	0	0	0	0	12	0	0	1
Chloromonadinia snow group 31	0	7	0	0	1	1	3	4
Chloromonadinia snow group 32	0	0	0	0	1	23	1	0
Chloromonadinia snow group 33	0	0	0	0	0	4	0	1
Chloromonadinia snow group 34	0	0	0	0	0	6	7	1
Chloromonadinia snow group 35	0	1	0	0	0	0	5	5
Chloromonadinia snow group 36	0	0	0	0	0	1	0	0
Chloromonadinia snow group 37	0	0	0	0	0	0	1	4
Chloromonadinia snow group 38	0	0	0	0	0	1	0	0
Chloromonadinia snow group 39	0	0	0	0	0	1	0	0
Chloromonadinia snow group 40	0	0	0	0	1	0	1	1
Chloromonadinia snow group 41	0	0	0	0	0	0	3	1
Chloromonadinia snow group 42	0	0	0	0	0	0	0	1
Chloromonadinia snow group 43	0	1	0	0	0	0	0	0
Chloromonadinia snow group 44	0	0	0	0	0	0	0	2
Raphidonema group	0	0	0	0	0	0	0	1
Pseudochlorella group	1	0	0	0	0	0	0	0
Apatococcus group	0	0	1	1	0	0	0	0
Dictyochloropsis group	1	0	1	1	0	0	0	0
Diplosphaera group	0	0	1	0	0	0	0	0
Myrmecia group	0	0	1	1	0	0	0	0
Neocystis group	0	0	1	0	0	0	0	0
Stichococcus group	1	1	1	1	0	0	0	0
Trebouxia CBC group 3	0	0	1	1	0	0	0	0
Trebouxia CBC group 4	1	0	0	0	0	0	0	0
Trebouxia CBC group 5	0	1	0	0	0	0	0	0
Trebouxia CBC group 7	2	0	1	1	0	0	0	0
Trebouxia CBC group 8	1	0	2	1	0	0	0	0
Trebouxia CBC group 9	2	0	11	7	2	0	0	0
Trebouxia CBC group 10	1	0	0	0	0	0	0	1
Trebouxia CBC group 11	0	0	0	1	0	0	0	0
Trebouxia CBC group 12	0	0	1	1	0	0	0	0
Total	10	21	22	16	18	47	183	89

Supplementary Table 8: Proportion of read sequences of ASVs detected in Hawai'i from the Antarctic or Arctic, Mid-Latitudes, and other regions.

ASVs	Taxonomy	Red Snow										NCBI nt database (100% similarity)
		Hawai'i	Antarctica	Svalbard	Alaska	Greenland	Russia	Japan	China	Tajikistan	Kyrgyz	
ASV_7	Sanguina group 2	5.98	0.0002	0.005	0.39	0.000	0.01	0	0	0.01	0	-
ASV_195	Sanguina group 2	0.17	0	0	0.0002	0	0	0	0	0	0	-
ASV_411	Sanguina group 2	0.06	0	0	0.0002	0	0	0	0	0	0	-
ASV_487	Sanguina group 2	0.05	0	0.0001	0.0002	0	0	0	0	0	0	-
ASV_144	Apatococcus group	0.27	-	-	-	-	-	-	-	-	-	Germany
ASV_1611	Pseudochlorella group	0.01	-	-	-	-	-	-	-	-	-	Antarctica (soil), USA, Germany
ASV_533	Trebouxia CBC group 1 (lichen)	0.04	0.02	0.0002	0.00	0	0.01	0	0	0	0	Poland, Sweden,
ASV_1417	Trebouxia CBC group 1 (lichen)	0.01	-	-	-	-	-	-	-	-	-	Bolivia
ASV_963	Trebouxia CBC group 5 (lichen)	0.01	-	-	-	-	-	-	-	-	-	Bolivia
ASV_873	Trebouxia CBC group 6 (lichen)	0.02	-	-	-	-	-	-	-	-	-	Bolivia, Costa Rica
ASV_657	Trebouxia CBC group 7 (lichen)	0.03	-	-	-	-	-	-	-	-	-	Chile, Spain
ASV_527	Trebouxia CBC group 8 (lichen)	0.04	0.001	0	0	0	0	0	0	0	0	Antarctica, Sweden, USA
ASV_2024	Trebouxia CBC group 8 (lichen)	0.002	-	-	-	-	-	-	-	-	-	USA, Argentina
ASV_24	Trebouxia CBC group 9 (lichen)	2.59	-	-	-	-	-	-	-	-	-	Antarctica, Norway, Turkey, USA
ASV_1215	Trebouxia CBC group 13 (lichen)	0.01	-	-	-	-	-	-	-	-	-	USA, Canada
